# Radical Stability Paradox: Substituent Effects *versus* Heats of Formation

**DOI:** 10.1002/chem.202503600

**Published:** 2025-12-28

**Authors:** Daniela Rodrigues Silva, Pascal Vermeeren, J. Martijn van der Schuur, F. Matthias Bickelhaupt

**Affiliations:** ^1^ Department of Chemistry and Pharmaceutical Sciences Amsterdam Institute of Molecular and Life Sciences (AIMMS) Vrije Universiteit Amsterdam Amsterdam The Netherlands; ^2^ Polymer Specialties Nouryon Deventer The Netherlands; ^3^ Institute of Molecules and Materials Radboud University Nijmegen The Netherlands; ^4^ Department of Chemical Sciences University of Johannesburg Johannesburg South Africa

**Keywords:** bonding theory, density functional calculations, radicals, substituent effects, stability

## Abstract

Recently, we have shown that alkyl substituents destabilize the carbon radical center of organic radicals. However, seemingly in contradiction with this earlier finding, the series of isomeric *n*‐butyl, *s*‐butyl, and *t*‐butyl radicals shows an increasingly stable (less positive) heat of formation ∆*H*_f_, despite an increasing number of alkyl substituents at the radical center. Herein, we provide a solution to this apparent paradox of contradicting pictures. The crux of the matter is that ∆*H*_f_ not only comprises the substituent effect on the radical center but also the intrinsic stability of the substituents. Furthermore, we provide a generalizable framework for extracting the actual intrinsic substituent effects from experimental and computational data, an approach with broad applicability to radical chemistry, thermochemistry, and beyond.

## Graphical abstract

**Rethinking molecular stability**: Quantum‐chemical analyses show that the greater stability of tertiary over primary organic radicals is not caused by stabilizing substituent effects but by the inherent stability of the substituent.

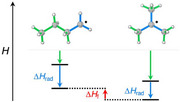

## 1 Introduction

Understanding the nature and stability of radicals helps to rationalize and tune the numerous reactions in which they feature as reactants, intermediates, and/or products. The stability of radicals is commonly quantified by the so‐called radical stabilization enthalpy (RSE) [1, 2]. For simple alkanes, the RSE is defined by the isodesmic reaction in Equation (1), which relates the homolytic C─H bond dissociation enthalpy of a given R_3_C–H molecule to that of the reference methane H_3_C–H molecule:

R_3_C^•^ + H_3_C–H → R_3_C–H + H_3_C^•^ Δ*H* = RSE (1)

It is known that the C─H bond in R_3_C–H becomes weaker, and the RSE becomes more positive, from methane to primary to secondary to tertiary alkanes [3, 4, 5]. Until recently, this trend was rationalized by the higher degree of substitution supposedly providing more stabilization to the R_3_C^•^ radical relative to the methyl radical (H_3_C^•^) because the alkyl groups delocalize the unpaired electron through hyperconjugation [6, 7, 8]. This explanation is still prevalent in many organic chemistry textbooks [9, 10, 11, 12], despite its validity being frequently questioned in the literature [13, 14, 15, 16, 17, 18, 19, 20, 21].

**FIGURE 1:**
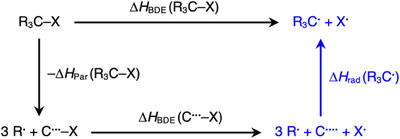
Thermodynamic cycle for the R_3_C─X bond dissociation (R_3_ = H_a_Me_b_Et_c_Pr_d_ with a+b+c+d = 3).

In 2022, Bickelhaupt and coworkers [22] showed that, in contrast with the textbook rationale, a higher degree of substitution *destabilizes*, not stabilizes, the R_3_C^•^ radical because C─C bonds are weaker than H─C bonds and because of a larger steric repulsion between alkyl than between hydrogen substituents. The abovementioned trend in R_3_C─H bond dissociation enthalpy depends on the substituent effect on *both* the radical R_3_C^•^ and the parent molecule R_3_C─H [Δ*H*_rad_(R_3_C^•^) *versus* Δ*H*_par_(R_3_C–H) in Figure 1]. This circumstance is often, if not always, overlooked in the interpretation of RSE trends. Thus, the reason why the corresponding C─H bond weakens upon introducing alkyl substituents is that alkyl substituents destabilize the sterically more congested parent molecule R_3_C–H to an even larger extent than the radical R_3_C^•^.

Another commonly used measure of stability is the heat of formation. Whereas the RSE is defined relative to a chosen reference molecule, the standard heat of formation Δ*H*_f_ is defined as the change in enthalpy associated with the formation of a compound from its respective elements in their natural state at standard temperature (25°C) and pressure (1 atm). For isomers, the elemental building blocks are the same, and therefore, Δ*H*_f_ is used to estimate the relative stabilities of these isomers [23]. For example, along a series of C_4_H_9_^•^ radical isomers, the Δ*H*_f_ values become less positive, that is, more stable, from primary *n*‐butyl (19.3 kcal mol^−1^) to secondary *s*‐butyl (16.5 kcal mol^−1^) to tertiary *t*‐butyl radical (11.5 kcal mol^−1^) (see Table S1) [24, 25, 26, 27, 28, 29, 30, 31]. This trend is seen as proof of the stabilizing effect of alkyl substituents on the radical centers, which confronts us with a paradox: Heats of formation ∆*H*_f_ indicate stabilization of the radical upon alkyl substitution, whereas stabilization by the substituents of the radical ∆*H*_rad_ indicate destabilization.

Herein, we resolve this paradox by reconciling the seemingly conflicting pictures of the substituent effect on the radical stability provided, on the one hand, by Δ*H*_rad_(R_3_C^•^), which shows that alkyl substituents destabilize the radical, and, on the other hand, the trends in the heat of formation Δ*H*_f_(R_3_C^•^), which become more stable if one goes to higher substituted radical isomers. Toward this goal, we have examined the substituent–carbon (R─C) bond in a more extensive set of R_3_C^•^ radicals (R_3_ = H_a_Me_b_Et_c_Pr_d_ with a+b+c+d = 3), which exhibit different numbers and different sizes of alkyl substituents. In our analyses, we use the activation strain model (ASM) [32, 33, 34] in conjunction with quantitative Kohn‐Sham molecular orbital (KS‐MO) [35, 36] theory and a matching energy decomposition analysis (EDA) [37, 38]. With the emerging insights, we analyze the formation of the series of C_4_H_9_^•^ radical isomers from their elemental building blocks using a thermochemical cycle, in which we relate Δ*H*_rad_(R_3_C^•^) with Δ*H*_f_(R_3_C^•^). We show again that, in all cases, alkyl substituents *destabilize* the radicals. Our analyses show that the heat of formation of R_3_C^•^ becomes nevertheless more stable (less positive) upon alkyl substitution, *e.g*., along *n‐*C_4_H_9_^•^, *s‐*C_4_H_9_^•^, *t‐*C_4_H_9_^•^, due to an increase in the substituents' intrinsic stability. The latter is key for understanding the trends in Δ*H*_f_(R_3_C^•^).

## 2 Results and Discussion

### 2.1 Substituent Effect

**FIGURE 2:**
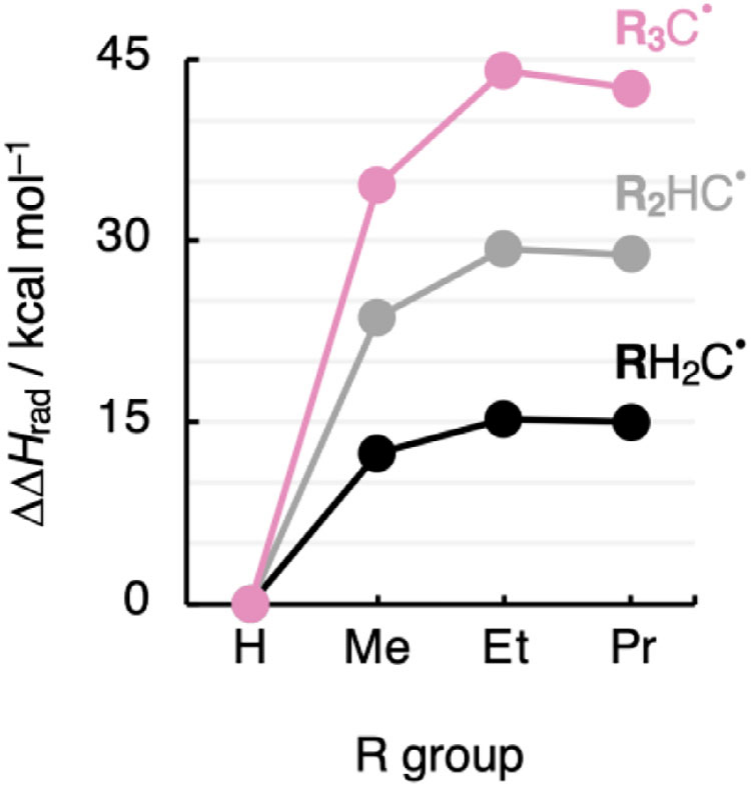
Substituent effect ∆Δ*H*_rad_ as a function of the number and size of substituents R^•^ in R_3_C^•^ radicals (R_3_ = H_a_Me_b_Et_c_Pr_d_ with a+b+c+d = 3), computed at UBLYP‐D3(BJ)/TZ2P for 298.15 K and 1 atm.

The substituent effect Δ*H*_rad_(R_3_C^•^) on the stability of the carbon radical center is computed according to the partial reaction of the thermochemical cycle shown in Figure 1, where Δ*H*_rad_(R_3_C^•^) is the overall bond enthalpy when the three separate substituents 3R^•^ combine with the carbon radical center C^••••^ to form the radical R_3_C^•^. The difference in stabilization by the substituents in R_3_C^•^ relative to the methyl radical H_3_C^•^ (ΔΔ*H*_rad_ in Equation (2)) as a function of the number and size of alkyl groups is plotted in Figure 2 (see also Figures S4–S5).

ΔΔ*H*_rad_ = Δ*H*_rad_(R_3_C^•^) − Δ*H*_rad_(H_3_C^•^) (2)

Figure 2 shows that alkyl substituents destabilize the radical center relative to hydrogen atoms (*i.e*., ΔΔ*H*_rad_ > 0). The destabilizing effect is approximately additive; that is, ΔΔ*H*_rad_ increases systematically by 10 to 15 kcal mol^−1^ as the number of alkyl R groups increases from one to two to three alkyl R groups (from black to gray to pink, respectively, in Figure 2). On the other hand, increasing the size of the substituents from R = Me to Et has a less pronounced destabilizing effect, and from R = Et to Pr, the effect is somewhat stabilizing (from left to right in Figure 2). Thus, where increasing the number of alkyl substituents results in significant destabilization of the radical, increasing the size of the alkyl substituents has a less pronounced destabilizing and eventually even a slightly stabilizing effect on the radical.

**FIGURE 3:**
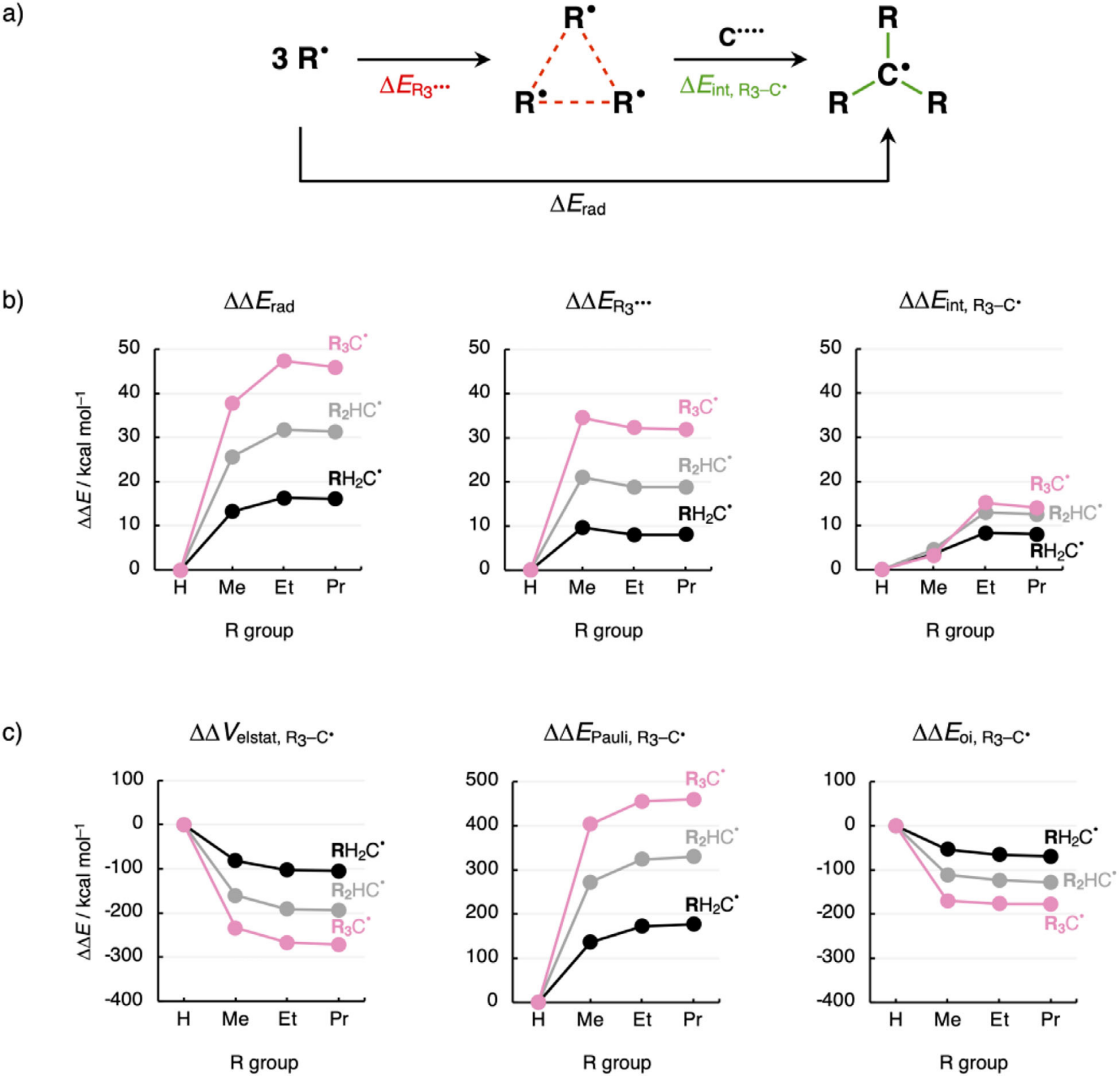
(a) Analyzing R_3_C^•^ in terms of two steps. (b) Substituent effect ∆Δ*E*_rad_ and its two components ∆∆*E*_R3•••_ and ∆∆*E*_int,R3–C•_ and (c) energy decomposition analysis (EDA) of the substituent‐effect component ∆*E*_int,R3–C•_ as a function of the number and size of substituents R^•^ in R_3_C^•^ radicals (R_3_ = H_a_Me_b_Et_c_Pr_d_ with a+b+c+d = 3), computed at UBLYP‐D3(BJ)/TZ2P. Dispersion energy ∆*E*_disp,R3–C•_ is nearly constant and, therefore, omitted as it does not affect the trends in interaction energy ∆∆*E*_int,R3–C•_ (see Figure S7).

To understand the trends in ΔΔ*H*_rad_, the bonding mechanism in the R_3_C^•^ radicals was analyzed in terms of two interacting fragments, namely, the cage of substituents R_3_^•••^ and the carbon radical center C^••••^ (Figure 3a; see the R–C electron‐pair bonds in Figure S6 and Table S2) [39, 40]. Note that the trend in ΔΔ*E*_rad_ determines in all cases the trend in ΔΔ*H*_rad_ (compare Figures 2 and 3b; see Theoretical Methods in the Supporting Information for details). Therefore, we analyze and decompose [32, 33, 34, 35, 36, 37, 38] ΔΔ*E*_rad_ into the corresponding difference in substituent energy ΔΔ*E*_R3•••_ and the difference in the interaction of the substituents R_3_^•••^ with the radical center C^••••^ ΔΔ*E*_int,R3–C•_ (Figure 3, all energy terms are relative to the methyl radical H_3_C^•^). Adding alkyl R groups destabilizes the system by both more destabilizing substituent energy (ΔΔ*E*_R3•••_ > 0) and less stabilizing interaction energy (ΔΔ*E*_int,R3–C•_ > 0; Figure 3b). This destabilizing effect arises from the increasing steric (Pauli) repulsion. As shown in previous works [4, 22], and further explained below, replacing a hydrogen atom (*i.e*., a C─H bond) with an alkyl group (*i.e*., a C─C bond) results in a weaker bond. The C─C bond connecting the alkyl substituent to the radical center is intrinsically weaker than the C─H bond because the additional carbon atom in the former has more closed electron shells than the hydrogen atom in the latter, which leads to more Paul repulsion, *i.e*., ΔΔ*E*_int,R3–C•_ > 0. Additionally, alkyl groups have more mutual steric Pauli repulsion among each other in R_3_^•••^ than hydrogen atoms, *i.e*., ΔΔ*E*_R3•••_ > 0. This repulsion increases as the number of alkyl R groups and their size increase.

The interaction energy between the cage of substituents and the carbon center (Δ*E*_int,R3–C•_) can be decomposed into four physically meaningful energy terms, namely, electrostatic interactions ΔΔ*V*_elstat,R3–C•_, steric (Pauli) repulsion Δ*E*_Pauli,R3–C•_, orbital interactions Δ*E*_oi,R3–C•_, and dispersion corrections Δ*E*_disp,R3–C•_ (a comprehensive overview of the method is provided in Theoretical Methods in the Supporting Information) [37, 38]. The enhanced steric (Pauli) repulsion upon alkyl substitution acts as an increasingly destabilizing term to the interaction energy Δ*E*_int,R3–C•_ (ΔΔ*E*_int,R3–C•_ > 0 and ΔΔ*E*_Pauli,R3–C•_ > 0 in Figures 3b and 3c, respectively). The number of closed‐shell subvalence orbitals, absent in H_3_^•••^, increases as R increases in size and, thus, provides an additional and evermore destabilizing contribution to Δ*E*_Pauli,R3–C•_ (left to right in Figure 3c; see Figure S8). Likewise, ΔΔ*E*_Pauli,R3–C•_ becomes systematically more destabilizing with the increasing degree of substitution (bottom to top in Figure 3c; see Supporting Discussion 1). Note that the electrostatic and orbital interactions (ΔΔ*V*_elstat,R3–C•_ and ΔΔ*E*_oi,R3–C•_) become more stabilizing upon alkyl substitution, thereby somewhat offsetting the ΔΔ*E*_Pauli,R3–C•_ for R = Pr (see Figure 3c).

**FIGURE 4:**
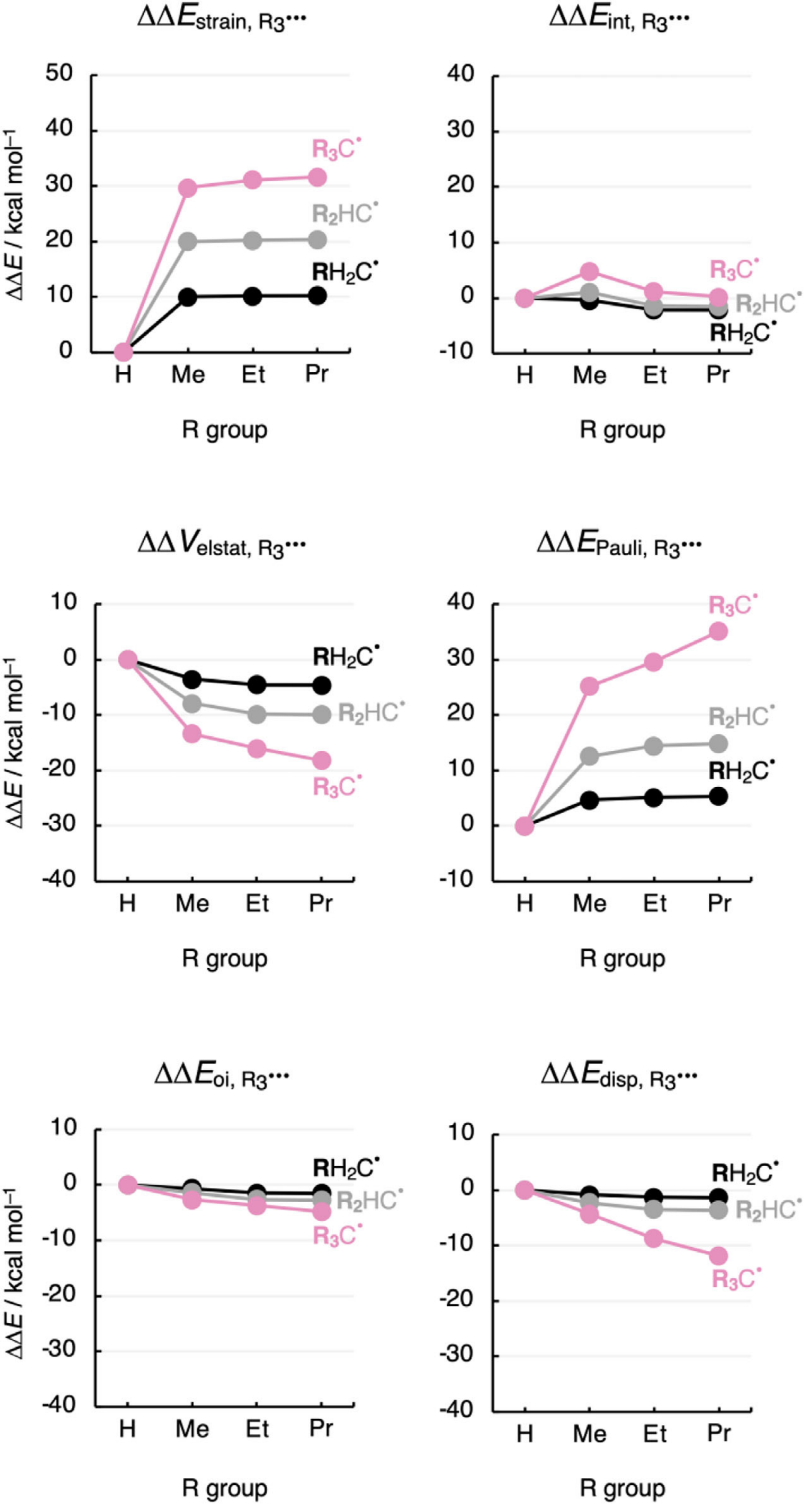
Activation strain model (ASM) and energy decomposition analysis (EDA) of the substituent energy ∆Δ*E*_R3•••_ as a function of the number and size of substituents R^•^ in R_3_C^•^ radicals (R_3_ = H_a_Me_b_Et_c_Pr_d_ with a+b+c+d = 3), computed at UBLYP‐D3(BJ)/TZ2P.

The increase in Pauli repulsion upon increasing the number and, to a lesser extent, the size of the substituents is also observed in the substituent energy ΔΔ*E*_R3•••_, which corresponds to building the cage of substituents R_3_^•••^ (in the geometry the cage adopts in R_3_C^•^) from three separate substituents R^•^, each one in its equilibrium geometry (Figure 3a). Alkyl substituents also experience more mutual steric (Pauli) repulsion than hydrogen atoms (ΔΔ*E*_Pauli,R3•••_ > 0; Figure 4). This term, ΔΔ*E*_Pauli,R3•••_, similarly to ΔΔ*E*_Pauli,R3–C•_, increases faster when increasing the number of substituents than when increasing the substituent size. Part of the enhanced steric repulsion is absorbed into the destabilizing strain (ΔΔ*E*_strain,R3•••_ > 0; Figure 4) of the substituent energy ΔΔ*E*_R3•••_ (see Theoretical Methods in the Supporting Information), which is associated with the geometrical deformation (*i.e*., pyramidalization), induced by the R─C bond formation and mutual repulsion between alkyl substituents (*ca*. 10 kcal mol^−1^ per R group) [39]. For larger R groups (R = Et, Pr), the increase in mutual steric repulsion is counterbalanced by increasingly stabilizing electrostatic interactions (ΔΔ*V*_elstat,R3•••_ < 0; Figure 4) to the extent that ΔΔ*E*_int,R3•••_ becomes somewhat more stabilizing from Me to Et to Pr. The Δ*V*_elstat,R3•••_ term is determined by the number of nuclei and electrons and how much they overlap (see Theoretical Methods in the Supporting Information). Thus, the nuclear–electron electrostatic attraction increases with the size of R. Furthermore, as expected for large systems, the dispersion energy ΔΔ*E*_disp,R3•••_ also counteracts the buildup of steric repulsion ΔΔ*E*_Pauli,R3•••_, although to a smaller extent than ΔΔ*V*_elstat,R3•••_.

In a nutshell, adding more substituents destabilizes the radical because C─C bonds are weaker than C─H bonds, and because alkyl groups experience greater steric repulsion than hydrogen atoms. However, increasing the size of the substituents from Me to Et to Pr has a less pronounced destabilizing effect. This is because longer substituents can engage in stabilizing interactions between their more remote sides (with less overlap but still electrostatic and dispersion interactions), referred to as steric attraction [41], that partially offset the increased steric Pauli repulsion, particularly when R = Pr.

### 2.2 Relating Substituent Effect to Heat of Formation

Now that we understand the substituent effect on the radical center given by Δ*H*_rad_(R_3_C^•^), we can analyze how it relates to the heat of formation Δ*H*_f_(R_3_C^•^). The seemingly contradictory trends in Δ*H*_rad_(R_3_C^•^) and Δ*H*_f_(R_3_C^•^) along primary *n*‐butyl to secondary *s*‐butyl to tertiary *t*‐butyl radical (*i.e*., increasingly destabilizing *versus* increasingly stable, *vide supra*) appear to originate from the circumstance that Δ*H*_f_(R_3_C^•^) ultimately informs one about the overall molecular stability, thus not only the effect of the R groups on the carbon radical center but also the intrinsic stability of the R groups. For example, for the series of C_4_H_9_^•^ radical isomers, Δ*H*_f_(R_3_C^•^) becomes less positive, *i.e*., more stable, from primary *n*‐butyl (19.3 kcal mol^−1^) to secondary *s*‐butyl (16.5 kcal mol^−1^) to tertiary *t*‐butyl radical (11.5 kcal mol^−1^) [24, 25, 26, 27, 28, 29, 30, 31] (see Table S1). In the following, we will explain that this is so because the substituents themselves become more stable along this series. Note that this occurs even though the effect of the alkyl groups on the carbon radical center C^••••^ becomes *less* stabilizing, not more stabilizing (Δ*H*_rad_(R_3_C^•^), *vide supra*).

**FIGURE 5:**
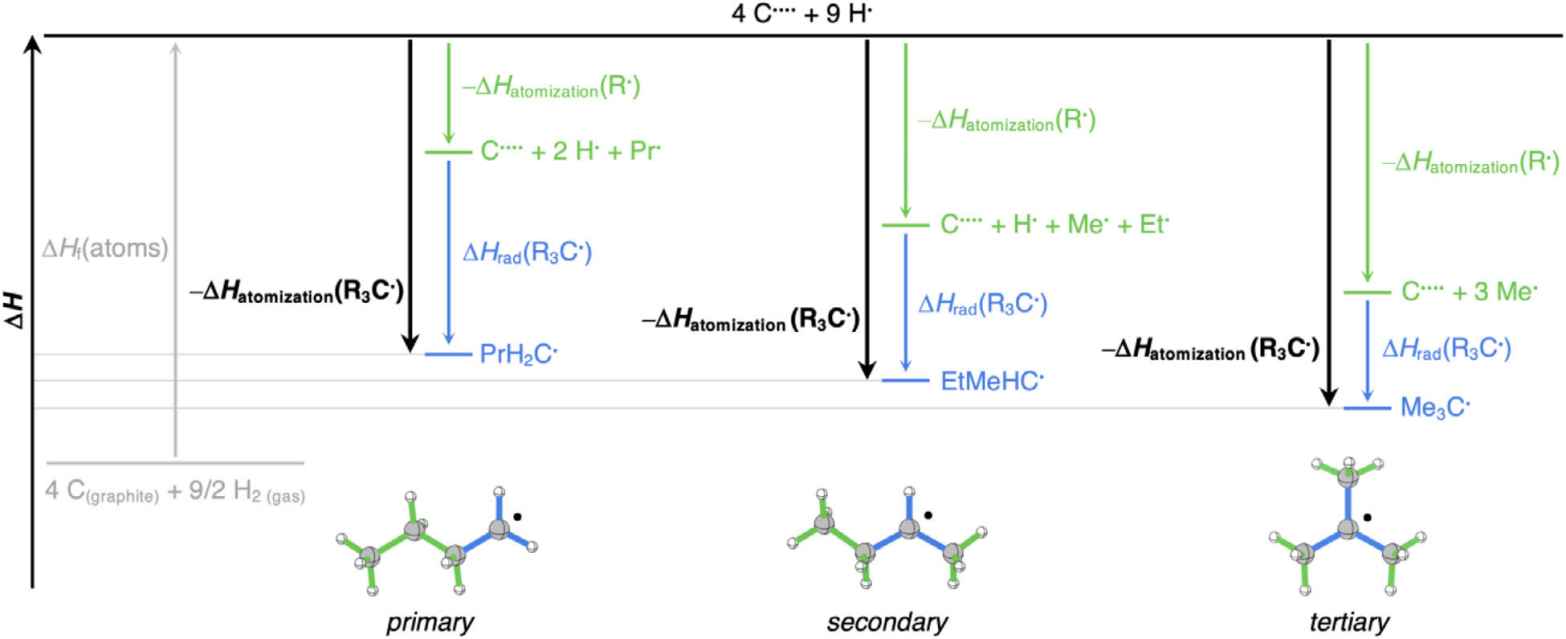
Thermochemical cycle of formation (in kcal mol^−1^) of the C_4_H_9_^•^ isomers of the R_3_C^•^ radicals (R_3_ = H_a_Me_b_Et_c_Pr_d_ with a+b+c+d = 3).

To understand the above trends, we have analyzed the formation of the C_4_H_9_^•^ radical isomers using the thermochemical cycle depicted in Figure 5 (all the computed bond enthalpies Δ*H* are provided in Table S1). First, there is the enthalpy associated with the formation of the substituents R^•^ from the atoms in their valence configuration (*i.e*., C^••••^ and H^•^), that is, the inverse of their atomization enthalpy: –Δ*H*_atomization_(R^•^) (green arrows in Figure 5). Next, three separate substituents 3R^•^ combine with C^••••^ to form each C_4_H_9_^•^ isomer (blue arrows in Figure 5), that is, the substituent effect on the radical R_3_C^•^ Δ*H*_rad_(R_3_C^•^), as shown in Figure 1. Thus, we have the relationship shown in Equation (3):

–Δ*H*_atomization_(R_3_C^•^) = –Δ*H*_atomization_(R^•^) + Δ*H*_rad_(R_3_C^•^) (3)

Δ*H*_f_(species) = –Δ*H*_atomization_(species) + Δ*H*_f_(atoms) (4)

Δ*H*_f_(atoms) = *n*Δ*H*_f_(H^•^) + *m*Δ*H*_f_(C^••••^) (5)

Herein, Δ*H*_atomization_(R_3_C^•^) (the inverse of the bold black arrows in Figure 5) is the enthalpy associated with the atomization of the R_3_C^•^ radical. It is related to Δ*H*_f_(R_3_C^•^) by the Δ*H*_f_(atoms) term in Equation (4), wherein “species” = R_3_C^•^ or R^•^. Δ*H*_f_(atoms) accounts for the heat of formation of the hydrogen and carbon atoms, that is, the atomization of C(graphite) and H_2_(gas) at standard temperature and pressure (25°C and 1 atm), and excitation of ground state C^••^ to valence state C^••••^ (Equation (5)). Note that, along a series of radical isomers, the term Δ*H*_f_(atoms) is constant and, therefore, shown in faded gray in the thermochemical cycle in Figure 5.

Although Δ*H*_f_(R_3_C^•^) contains Δ*H*_rad_(R_3_C^•^) (Equations (3) and (4)), Δ*H*_rad_(R_3_C^•^) does not explain the trends in Δ*H*_f_(R_3_C^•^). In contrast to Δ*H*_f_(R_3_C^•^), Δ*H*_rad_(R_3_C^•^) is destabilized if one goes from primary (–389.4 kcal mol^−1^) to secondary (–378.0 kcal mol^−1^) to tertiary radical isomers (–369.9 kcal mol^−1^) (*vide supra*). The trends in Δ*H*_f_ are recovered only when the stability of the substituents R, as contained in –Δ*H*_atomization_(R^•^), is also included. The substituents become more stable from primary to secondary to tertiary radicals as follows from –Δ*H*_atomization_(R^•^), which varies from –1185.7 kcal mol^−1^ to –1201.0 kcal mol^−1^ to –1213.5 kcal mol^−1^ along this series. The origin of this trend is the ratio *C–H*: *C–C* within the substituents, *i.e*., the ratio between the number of C─H bonds (which are stronger) and C─C bonds (which are weaker). This *C–H: C–C* ratio increases from *7*:*2* to *8*:*1* to *9*:*0* along the primary, secondary, and tertiary C_4_H_9_^•^ radical isomers. Importantly, however, –Δ*H*_atomization_(R^•^) is stabilized more than Δ*H*_rad_(R_3_C^•^) is destabilized along this series. Thus, the fact that the substituents become more stable from primary to secondary to tertiary radicals because they have stronger bonds (*i.e*., more C─H bonds) causes Δ*H*_f_(R_3_C^•^) to become more stable (*i.e*., less positive) along this series, although the substituents stabilize the radical less. The weakening of bonds to the radical center *versus* the strengthening of bonds within the substituents apparently does not cancel each other out. In the next section, we address this situation in more detail.

### 2.3 Heats of Formation

The question now is: Why, along the primary, secondary, and tertiary C_4_H_9_^•^ radical isomers, do we gain more in intrinsic substituent stability –Δ*H*_atomization_(R^•^) than we lose in stabilization by the substituents of the radical ∆*H*_rad_(R_3_C^•^) or, in other words, why does the heat of formation Δ*H*_f_(R_3_C^•^) become more stable (*i.e*., less positive) whereas the total number of C–H and the total number of C─C bonds remain the same? The answer is rather simple: C─H bonds differ in strength in the different isomers, and so do the C─C bonds (Figure S9). As will become clear in the following, the C─H bonds are strongest at primary carbons and the C─C bonds are strongest at three‐coordinate carbon radical centers. This implies that the *t*‐butyl radical is the most stable C_4_H_9_^•^ isomer.

Two distinct factors can affect the strength of C–H and C─C bonds (Figure S10), albeit to a different extent for the former compared to the latter. The first one is the number of substituents, *i.e*., the coordination number n+1 around the pertinent central carbon atom in an R_n_C─H bond or an R_n_C─C bond. The second factor is the steric size of the groups R attached to the central carbon atom, that is, hydrogen atoms as, for example, in the unsubstituted H_3_C–H or H_3_C─C bond, or an alkyl group as, for example, in the methyl‐substituted Me_3_C–H or Me_3_C─C bond. *A priori*, one can expect that increasing the coordination number, *e.g*., from n+1 = 3 to 4, is a drastic change in steric crowding and will go with a significant destabilization of the R_n_C─H bond and, even more so, the R_n_C─C bond as this latter involves an additional alkyl substituent. Increasing the steric size of substituents R involved in R_n_C–H and R_n_C─C bonds is also expected to raise the steric repulsion and thus destabilize the bonds. However, the relative magnitude of the destabilizing effect on C–H *versus* C─C bonds is more difficult to predict qualitatively. The C─C bond might experience a larger increase in Pauli repulsion than the C–H due to the additional alkyl substituent. But the C─C bond is also longer than the C─H bond, and the destabilizing Pauli effects might be mitigated by more stabilizing dispersion and/or electrostatic attraction with this additional alkyl substituent. Our quantitative analyses reveal that increasing the coordination number indeed destabilizes the C─C bond *more* than the C─H bond, whereas increasing the size of alkyl substituents destabilizes C─C bonds *less* than C─H bonds. This makes the heat of formation more stable (less positive) for the more branched isomers.

As previously mentioned, increasing the coordination number in R_n_C–H and R_n_C–C from n + 1 = 3 to 4 leads to a weakening of both C–H and C─C bonds due to increased steric congestion around the central carbon [4]. For example, the C─H bond strength decreases from Δ*H*_bond_ = –111.0 kcal mol^−1^ for methyl (H_2_C^•^–H) to Δ*H*_bond_ = –102.7 kcal mol^−1^ for methane (H_3_C–H), whereas the C─C bond strength decreases from Δ*H*_bond_ = –98.5 kcal mol^−1^ for ethyl (H_2_C^•^–CH_3_) to Δ*H*_bond_ = –85.2 kcal mol^−1^ for ethane (H_3_C–CH_3_) (Table S3). The destabilizing effect of increasing the coordination number is, however, more pronounced for the C─C bond, which weakens by 13.3 kcal mol^−1^, compared to 8.3 kcal mol^−1^ for the C─H bond. This is because the C─C bond experiences greater steric repulsion, due to the larger number of closed‐shell orbitals in ^•^CH_3_ compared to ^•^H (Figure S11). As a result, the strength of the C─C bond is more sensitive to changes in coordination number. Therefore, radical isomers featuring C─C bonds at low‐coordination carbon centers, *i.e*., at the radical center, have stronger bonds and are consequently more stable.

Similarly, increasing the steric size of the R groups attached to the central carbon in R_n_C─H and R_n_C–C (n + 1 = 3 or 4) weakens the corresponding bonds due to enhanced steric repulsion (Table S3) [4, 22]. For example, the C─H bond strength weakens from Δ*H*_bond_ = –102.7 kcal mol^−1^ for methane (H_3_C–H) to Δ*H*_bond_ = –91.2 kcal mol^−1^ for 2‐methylpropane (Me_3_C–H), whereas the C─C bond strength weakens from Δ*H*_bond_ = –85.2 kcal mol^−1^ for ethane (H_3_C–CH_3_) to Δ*H*_bond_ = –78.0 kcal mol^−1^ for 2,2‐dimethylpropane (Me_3_C–CH_3_) (Figure S12 and Table S3). In this case, however, the C─C bond is less sensitive to the increasing steric size of the groups R than the C─H bond, with bond strength weakening of 7.2 and 11.5 kcal mol^−1^, respectively. Although the C─C bond experiences greater steric repulsion due to more closed‐shell orbitals, as discussed above, it also benefits from stabilizing interactions that favor steric crowding at constant coordination number. These steric‐attraction effects partially offset the destabilizing steric Pauli repulsion, resulting in a smaller overall weakening of the C─C bond than might be expected based on steric Pauli repulsion alone (Supporting Discussion 2). Consequently, the C─H bond is more sensitive to the steric size of the R groups, and isomers featuring C─H bonds with fewer geminal alkyl substituents, *i.e*., those located on primary carbons, are therefore more stable. As a result, along the C_4_H_9_^•^ radical series, both C─H and C─C bonds become progressively stronger when moving from primary to secondary to tertiary isomers. This trend arises from the decreasing steric size of the substituents around the C─H bonds and the lowering of the coordination number around the C─C bonds along the series (Supporting Discussion 3 and Figure S13).

The same rationale explains why more branched alkanes have a more stable heat of formation than linear ones, a subject of a long‐lasting debate in the literature [17, 42, 43, 44, 45, 46, 47, 48, 49, 50, 51, 52, 53]. Let us take the C_4_H_10_ alkane isomers associated with the C_4_H_9_^•^ radicals depicted in Figure 5 as an example (Supporting Discussion 4, Figure S14, and Table S4). The C_4_H_10_ alkane isomers become more stable from butane to isobutane (Δ*H*_f_(R_3_C–H) = –30.0 and –32.1 kcal mol^−1^, respectively) [24, 25, 26, 27, 28, 29, 30, 31], that is, from primary to tertiary, or from linear to branched alkane. As the coordination number does not change in this case (*i.e*., n+1 = 4), this trend in Δ*H*_f_ originates entirely from the steric size of the groups R around the C─H bond. Therefore, the more branched isomer is more stable despite having a sterically more crowded carbon center because it maximizes the number of less sterically hindered (*i.e*., stronger) C─H bonds in primary carbons (Supporting Discussion 4 and Figure S15).

In essence, Δ*H*_f_ ultimately informs about the strength of all chemical bonds within a molecule together, which become, on average, stronger going from primary to secondary to tertiary isomers. The current rationale that alkyl substituents stabilize organic radicals is a result of a misinterpretation of Δ*H*_f_ as well as of RSE values [22]. Both Δ*H*_f_ and RSE values (see Table S5) depend not solely on the actual substituent effects. Hence, their value and trends therein must be interpreted with caution. The Δ*H*_rad_ value, however, directly quantifies the actual effect of substituents on any radical. In the following section, we show how this actual substituent effect Δ*H*_rad_ can be calculated not only from computational but also from experimental data.

### 2.4 Calculating the Actual Substituent Effect

**FIGURE 6:**
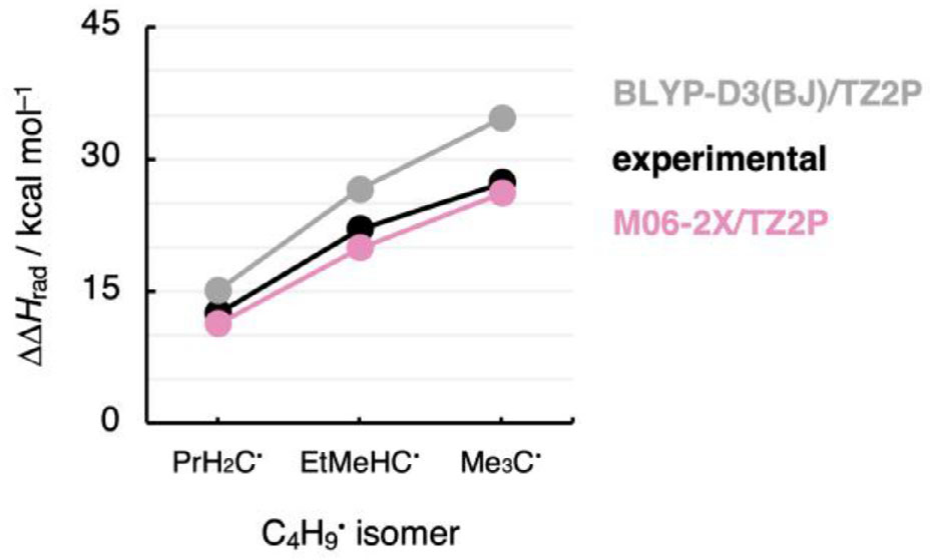
Comparison of stabilization by the substituents of the radical relative to the methyl radical ΔΔ*H*_rad_ computed quantum chemically at UDFT/TZ2P (DFT = BLYP‐D3(BJ) and M06‐2X) and calculated from the experimental heats of formation Δ*H*_f_ for the C_4_H_9_^•^ isomers.

According to Figure 1, Δ*H*_rad_(R_3_C^•^) can be readily calculated by Equation (6) using also experimental data if the experimental Δ*H*_f_ of the radical and its corresponding fragments (*e.g*., R_3_C^•^ and the R^•^s) are available. Figure 6 compares ΔΔ*H*_rad_ calculated from quantum chemical computations and experimentally obtained Δ*H*_f_ data for the series of C_4_H_9_^•^ radical isomers; data for other R_3_C^•^ radicals are shown in Table S1. The theoretically and experimentally obtained ΔΔ*H*_rad_ differ by only a few kcal mol^−1^, and this small difference can be attributed to both uncertainties in the experimental measurement [24, 25, 26, 27, 28, 29, 30, 31] and the level of theory used (see Table S1). Notably, the qualitative picture is *exactly* the same: alkyl substituents *destabilize* radicals, and the effect of increasing the degree of substitution is larger than the effect of going from shorter to longer substituents.

Δ*H*_rad_(R_3_C^•^) = Δ*H*_f_(R_3_C^•^) – 3 Δ*H*_f_(R^•^) − Δ*H*_f_(C^••••^) (6)

## 3 Conclusion

We resolve a seemingly paradoxical trend. Recently, we showed that alkyl substitution destabilizes organic radicals. However, seemingly in contradiction to this finding, the enthalpy of formation ∆*H*_f_(R_3_C^•^) of the *n*‐butyl, *s*‐butyl, and *t*‐butyl radical isomers decreases along that series, that is, as the degree of substitution *increases* from primary to secondary to tertiary isomers. The conventional textbook explanation for this trend posits that substituting hydrogen for alkyl groups stabilizes the radical center by delocalizing the unpaired electron through hyperconjugation. However, we show in line with earlier work that this rationale is erroneous. The predominant effect of such substitution, for instance, from H_3_C^•^ to Me_3_C^•^, is really a destabilization of the radical center. The reason is that C─C bonds are weaker than C─H bonds, and alkyl substituents have more mutual repulsion. This is shown by the stabilization by the substituents of the radical ∆*H*_rad_(R_3_C^•^), which becomes less stabilizing from R = H to R = Me or other alkyl substituents.

The resolution to the paradox above lies in the distinction that the heat of formation Δ*H*_f_(R_3_C^•^) = Δ*H*_rad_(R_3_C^•^)) + 3 Δ*H*_f_(R^•^) + Δ*H*_f_(C^••••^) incorporates the intrinsic stability of the substituents whereas, in contrast, the pure stabilization by the substituents ∆*H*_rad_(R_3_C^•^) does not. Therefore, the heat of formation ∆*H*_f_(R_3_C^•^) can become more stable if the intrinsic stability of the substituents increases, even if simultaneously ∆*H*_rad_(R_3_C^•^) becomes less stabilizing.

Finally, our analyses show that (and why) the most stable alkyl radical isomer arises from maximizing (i) the number of C─H bonds at primary carbons and (ii) the number of C─C bonds at the carbon radical center. The same holds *mutatis mutandis* for the corresponding alkanes: the most stable alkane isomer has more C─H bonds at primary carbons [as there exists no radical center in an alkane, factor (ii) does not apply]. This explains why along the isomeric series *n*‐butyl to *s*‐butyl to *t*‐butyl radical Δ*H*_f_(R_3_C^•^) is stabilized, not constant (and also not destabilized), even though the total number of C─H bonds and C─C bonds is conserved.

## Conflicts of Interest

There are no conflicts of interest to declare.

## Supporting information




**Supporting File 1**: chem70635‐sup‐0001‐SuppMat.pdf
