# Supplementary material for: Radical Stability Paradox: Substituent Effects versus Heats of Formation
Source: Chemistry. 2025 Dec 28;32(7):e03600. doi: 10.1002/chem.202503600 (PMC12910414; doi:10.1002/chem.202503600)
Supplement: Supplementary file 1 — Supporting File 1: chem70635‐sup‐0001‐SuppMat.pdf [file CHEM-32-e03600-s001.pdf]

Table of Contents

Theoretical Methods S3

Table S1. Energy of formation of radicals S7

Figure S1. Effect of the degree of alkyl substitution and size S8

Figure S2. Radical energy analysis S8

Figure S3. Decomposition of the substituent energy S9

Figure S4. Effect of the degree of alkyl substitution and size S10

Figure S5. Geometrical data S10

Figure S6. Schematic representation of the R_3–$C^•$ electron-pair bonds S11

Table S2. Electron-pair bonds S12

Figure S7. Decomposition of the interaction energy $\Delta E_{int,R_3-C^•}$ S12

Figure S8. Orbital overlap matrix S13

Supporting Discussion 1. Pauli repulsion S14

Figure S9. Bond strength S15

Figure S10. Effect of the coordination number and substituent steric size S15

Supporting Discussion 2. Strength of C–H and C–C bonds S16

Table S3. Factors affecting the strength of C–H and C–C bonds S16

Figure S11. Effect of the coordination number S17

Figure S12. Effect of the steric size of the substituents S17

Supporting Discussion 3. Heat of formation of the $R_3C^•$ radicals S18

Figure S13. Stepwise formation of radical isomers S18

Supporting Discussion 4. Heat of formation of the R_3C–H parent molecules S20

Figure S14. Enthalpy of formation of alkane isomers S20

Table S4. Energy of formation of alkanes S21

Figure S15. Stepwise formation of alkane isomers S22

Table S5. Experimental and computational enthalpies of the $C_4H_9^•$ radical isomers S23

Supporting Data 1. Cartesian coordinates S24

Table S6. Cartesian coordinates of radicals at UBLYP-D3(BJ) S24

Table S7. Cartesian coordinates of alkanes at BLYP-D3(BJ) S33

Table S8. Cartesian coordinates of radicals at UM06-2X S38

Table S9. Cartesian coordinates of alkanes at M06-2X S44

Theoretical Methods

Computational details

All calculations were performed using the Amsterdam Density Functional (ADF) program (ADF2019.305).[1–3] Molecular orbitals (MOs) were expanded using a large, uncontracted set of Slater-type orbitals (STO): TZ2P.[4] The TZ2P basis set is of triple-ζ quality, augmented by two sets of polarization functions, *i.e.*, 2*p* and 3*d* on H and 3*d* and 4*f* on C. All electrons were treated variationally. Geometries and energies were calculated at the BLYP level of the generalized gradient approximation (GGA), the exchange functional developed by Becke (B), and the GGA correlation functional developed by Lee, Yang, and Parr (LYP).[5,6] The DFT-D3(BJ) correction developed by Grimme and coworkers,[7,8] which contains the damping function introduced by Becke and Johnson,[9] was used to account for dispersion interactions. All trends and conclusions emerging from our BLYP-D3(BJ)/TZ2P computations are fully reproduced at the M06-2X[10]/TZ2P level (see Figures S1–S3). Note that there is a somewhat better agreement of M06-2X than BLYP-D3(BJ) with experimental data (see Table S1). However, BLYP-D3(BJ) enables us to estimate the importance of dispersion interactions (see Radical energy analysis section below) and is, therefore, employed in our bonding analyses presented in this manuscript, whereas the M06-2X data is provided in the Supporting Information. The numerical accuracy was set to EXCELLENT.[11,12] All optimized structures were confirmed to be true minima (no imaginary frequencies) through vibrational analyses.[13–15] Radical species were treated with a spin-unrestricted formalism. All structures were illustrated using CYLview.[16]

Conformational search

The conformational space of the $R_3C^{\bullet}$ radicals and the corresponding parent molecule R_3C–H (R_3 = $H_aMe_bEt_cPr_d$ with a+b+c+d = 3) in the gas phase was screened using the Conformer–Rotamer Ensemble Sampling Tool (CREST),[17] which combines a metadynamics search algorithm[18] with semiempirical tight-binding GFN*n*-xTB methods.[19] The lowest energy conformations were reoptimized using UDFT/TZ2P with DFT = BLYP-D3(BJ) and M06-2X. The global minimum energy conformation was used in all further analyses.

Thermochemistry

Enthalpies at 298.15 K and 1 atmosphere (ΔH) were calculated from electronic bond energies (ΔE) and vibrational frequencies using standard thermochemistry relations for an ideal gas, according to Equation (S1):[20,21]

$$\Delta H = \Delta E + \Delta E_{\mathrm{trans,298}} + \Delta E_{\mathrm{rot,298}} + \Delta E_{\mathrm{vib,0}} + \Delta(\Delta E_{\mathrm{vib,0}})_{298} + \Delta(pV) \qquad \text{(S1)}$$

Here, $\Delta E_{\text{trans},298}$, $\Delta E_{\text{rot},298}$, and $\Delta E_{\text{vib},0}$ are the differences between the separate fragments and the combined species in translational, rotational, and zero-point vibrational energy, respectively. $\Delta(\Delta E_{\text{vib},0})_{298}$ is the change in the vibrational energy difference as one goes from 0 to 298.15 K. The vibrational energy corrections are based on our frequency calculations. Thermal corrections for the electronic energy are neglected.

Radical energy analysis

The bonding mechanism in the $R_3C^{\bullet}$ radicals ($R_3 = H_aMe_bEt_cPr_d$ with a+b+c+d = 3) was analyzed in terms of two interacting fragments, $R_3^{\bullet\bullet\bullet}$ and $C^{\bullet\bullet\bullet\bullet}$.[22,23] The radical bond energy ΔE_{rad} is divided into two components as in Equation (S2):

$$\Delta E_{\text{rad}} = \Delta E_{R_3^{\bullet\bullet\bullet}} + \Delta E_{\text{int},R_3\text{–}C^{\bullet}} \tag{S2}$$

Here, the substituent energy $\Delta E_{R_3^{\bullet\bullet\bullet}}$ corresponds to the formation of the $R_3^{\bullet\bullet\bullet}$ fragment in its quartet valence configuration and in the geometry which it acquires in the final $R_3C^{\bullet}$ radical, and the interaction energy $\Delta E_{\text{int},R_3\text{–}C^{\bullet}}$ is the actual energy change when the carbon atom in its valence $2s^12p^3$ configuration $C^{\bullet\bullet\bullet\bullet}$ and the prepared $R_3^{\bullet\bullet\bullet}$ fragment are combined to form $R_3C^{\bullet}$ (Figure 3a).

The substituent energy $\Delta E_{R_3^{\bullet\bullet\bullet}}$ can be further decomposed using the activation strain model (ASM)[24–26] into the corresponding strain energy $\Delta E_{\text{strain},R_3^{\bullet\bullet\bullet}}$ and interaction energy $\Delta E_{\text{int},R_3^{\bullet\bullet\bullet}}$ (Equation (S3)):

$$\Delta E_{R_3^{\bullet\bullet\bullet}} = \Delta E_{\text{strain},R_3^{\bullet\bullet\bullet}} + \Delta E_{\text{int},R_3^{\bullet\bullet\bullet}} \tag{S3}$$

The strain energy $\Delta E_{\text{strain},R_3^{\bullet\bullet\bullet}}$ is the amount of energy required to deform the $R^{\bullet}$ fragments from their equilibrium structure to the geometry that they acquire in the final $R_3C^{\bullet}$ radical. The interaction energy $\Delta E_{\text{int},R_3^{\bullet\bullet\bullet}}$ is the actual energy change when the deformed $R^{\bullet}$ fragments are combined to form $R_3^{\bullet\bullet\bullet}$.

We further analyze any interaction energy ΔE_{int} within the framework of the Kohn-Sham molecular orbital (KS-MO) theory[27,28] by dissecting it using our canonical energy decomposition analysis (EDA) scheme into electrostatic interactions, Pauli repulsive orbital interactions, stabilizing orbital interactions, and dispersion corrections (Equation (S4)):[29,30]

$$\Delta E_{\text{int}} = \Delta V_{\text{elstat}} + \Delta E_{\text{Pauli}} + \Delta E_{\text{oi}} + \Delta E_{\text{disp}} \tag{S4}$$

The electrostatic energy ΔV_{elstat} corresponds to the electrostatic interactions between the unperturbed charge distribution of the fragments, which is usually attractive. The ΔV_{elstat} term can be further divided into four components (Equation (S5)):

$$\Delta V_{\mathrm{elstat}} = \sum_{\substack{\alpha \in A \\ \beta \in B}} \frac{Z_\alpha Z_\beta}{R_{\alpha\beta}} - \int \sum_{\alpha \in A} \frac{Z_\alpha \rho_B(r)}{|r - R_\alpha|} \mathrm{d}r - \int \sum_{\beta \in B} \frac{Z_\beta \rho_A(r)}{|r - R_\beta|} \mathrm{d}r + \int\int \frac{\rho_A(r_1)\rho_B(r_2)}{r_{12}} \mathrm{d}r_1 \mathrm{d}r_2$$

$$\Delta V_{\mathrm{elstat}} = \Delta V_{\mathrm{elstat,\ N\text{–}N}} + \Delta V_{\mathrm{elstat,\ N\text{–}e}} + \Delta V_{\mathrm{elstat,\ e\text{–}N}} + \Delta V_{\mathrm{elstat,\ e\text{–}e}} \quad \text{(S5)}$$

The first term in Equation (S5) is the electrostatic repulsion between the nuclei n of fragments A and B (A = $R_3^{\bullet\bullet\bullet}$ and B = $C^{\bullet\bullet\bullet\bullet}$), $\Delta V_{\mathrm{elstat,N\text{–}N}}$; the second and third terms are the electrostatic attraction between the nuclei n of fragment A and the electron density ρ of fragment B, $\Delta V_{\mathrm{elstat,N\text{–}e}}$, and vice versa, $\Delta V_{\mathrm{elstat,e\text{–}N}}$; while the last term is the electrostatic repulsion between the electron densities ρ of fragments A and B, $\Delta V_{\mathrm{elstat,e\text{–}e}}$.

The Pauli repulsion $\Delta E_{\mathrm{Pauli}}$ comprises the destabilizing interactions between occupied same-spin orbitals and is responsible for any steric repulsion. The orbital interactions ΔE_{oi} term accounts for electron-pair bonding (the SOMO–SOMO interaction), charge transfer (donor–acceptor interaction between an occupied orbital of one fragment with an empty orbital of the other fragment), and polarization (empty/occupied orbital mixing on one fragment due to the presence of another fragment). Finally, the dispersion energy ΔE_{disp} is added as a correction using the approach of Grimme and coworkers.[7,8]

References

[1] *ADF2019.3*, SCM, Theoretical Chemistry, Vrije Universiteit Amsterdam, Amsterdam, The Netherlands, http://www.scm.com.

[2] G. te Velde, F. M. Bickelhaupt, E. J. Baerends, C. Fonseca Guerra, S. J. A. van Gisbergen, J. G. Snijders, T. Ziegler, *J. Comput. Chem.* **2001**, *22*, 931–967.

[3] C. Fonseca Guerra, J. G. Snijders, G. te Velde, E. J. Baerends, *Theor. Chem. Acc.* **1998**, *99*, 391–403.

[4] E. van Lenthe, E. J. Baerends, *J. Comput. Chem.* **2003**, *24*, 1142–1156.

[5] A. D. Becke, *Phys. Rev. A* **1988**, *38*, 3098–3100.

[6] C. T. Lee, W. T. Yang, R. G. Parr, *Phys. Rev. B* **1988**, *37*, 785–789.

[7] S. Grimme, J. Antony, S. Ehrlich, H. Krieg, *J. Chem. Phys.* **2010**, *132*, 154104.

[8] S. Grimme, S. Ehrlich, L. Goerigk, *J. Comput. Chem.* **2011**, *32*, 1456–1465.

[9] E. R. Johnson, A. D. Becke, *J. Chem. Phys.* **2005**, *123*, 024101.

[10] Y. Zhao, D. G. Truhlar, *Theor. Chem. Acc.* **2008**, *120*, 215–241.

[11] M. Franchini, P. H. T. Philipsen, E. van Lenthe, L. Visscher, *J. Chem. Theory Comput.* **2014**, *10*, 1994–2004.

[12] M. Franchini, P. H. T. Philipsen, L. Visscher, *J. Comput. Chem.* **2013**, *34*, 1819–1827.

[13] A. Bérces, R. M. Dickson, L. Fan, H. Jacobsen, D. Swerhone, T. Ziegler, *Comput. Phys. Commun.* **1997**, *100*, 247–262.

[14] H. Jacobsen, A. Bérces, D. P. Swerhone, T. Ziegler, *Comput. Phys. Commun.* **1997**, *100*, 263–276.

[15] S. K. Wolff, *Int. J. Quantum Chem.* **2005**, *104*, 645–659.

[16] C. Y. Legault, *CYLview20*, Université de Sherbrooke, Sherbrooke, Quebec, Canada, **2020**, www.cylview.org.

[17] P. Pracht, F. Bohle, S. Grimme, *Phys. Chem. Chem. Phys.* **2020**, *22*, 7169–7192.

[18] S. Grimme, *J. Chem. Theory Comput.* **2019**, *15*, 2847–2862.

[19] C. Bannwarth, E. Caldeweyher, S. Ehlert, A. Hansen, P. Pracht, J. Seibert, S. Spicher, S. Grimme, *WIREs Comput. Mol. Sci.* **2020**, *11*, e01493.

[20] P. W. Atkins, J. de Paula, *Physical Chemistry,* 9th ed., W. H. Freeman, New York, **2010**.

[21] F. Jensen, *Introduction to Computational Chemistry*, Wiley, **2007**.

[22] F. M. Bickelhaupt, T. Ziegler, P. von Ragué Schleyer, *Organometallics* **1996**, *15*, 1477–1487.

[23] D. Rodrigues Silva, L. de Azevedo Santos, M. P. Freitas, C. Fonseca Guerra, T. A. Hamlin, *Chem. Asian. J.* **2020**, *15*, 4043–4054.

[24] P. Vermeeren, T. A. Hamlin, F. M. Bickelhaupt, *Chem. Comm.* **2021**, *57*, 5880–5896.

[25] P. Vermeeren, S. C. C. van der Lubbe, C. Fonseca Guerra, F. M. Bickelhaupt, T. A. Hamlin, *Nat. Protoc.* **2020**, *15*, 649–667.

[26] F. M. Bickelhaupt, K. N. Houk, *Angew. Chem. Int. Ed.* **2017**, *56*, 10070–10086; *Angew. Chem.* **2017**, *129*, 10204–10221.

[27] R. van Meer, O. V. Gritsenko, E. J. Baerends, *J. Chem Theory Comput.* **2014**, *10*, 4432–4441.

[28] T. A. Albright, J. K. Burdett, W. H. Wangbo, *Orbital Interactions in Chemistry*, Wiley, Hoboken, **2013**.

[29] T. A. Hamlin, P. Vermeeren, C. Fonseca Guerra, F. M. Bickelhaupt, *Complementary Bonding Analysis* Ch. 8, ed. S. Grabowski, De Gruyter, Berlin, **2021**.

[30] F. M. Bickelhaupt, E. J. Baerends, *Reviews in Computational Chemistry* Ch. 1, ed. K. B. Lipkowitz, D. B. Boyd, Wiley–VCH, New York, **2000**.

Table S1. Electronic energies and enthalpies (in kcal mol^{-1}) of the $R_3C^{\bullet}$ radicals ($R_3 = H_aMe_bEt_cPr_d$ with a+b+c+d = 3) according to the thermochemical cycle in Figure 5.[a, b]

Systems[b]	$-\Delta E_{atom}$ ($R_3C^{\bullet}$)	$-\Delta E_{atom}$ ($R^{\bullet}$)	ΔE_{rad} ($R_3C^{\bullet}$)	$-\Delta H_{atom}$ ($R_3C^{\bullet}$)	$-\Delta H_{atom}$ ($R^{\bullet}$)	ΔH_{rad} ($R_3C^{\bullet}$)	ΔH_f ($R_3C^{\bullet}$)[c]	ΔH_{rad} ($R_3C^{\bullet}$)[e]
**$H_3C^{\bullet}$**	–419.3	0.0	–419.3	–404.5	0.0	–404.5	35.1	–388.8
	(–414.8)	(0.0)	(–414.8)	(–399.5)	(0.0)	(–399.5)		
**$MeH_2C^{\bullet}$**	–825.3	–419.3	–406.0	–796.5	–404.5	–392.0	28.4	–378.6
	(–819.5)	(–414.8)	(–404.6)	(–789.2)	(–399.5)	(–389.7)		
**$EtH_2C^{\bullet}$**	–1228.3	–825.3	–403.0	–1185.7	–796.5	–389.2	23.9	–376.4
	(–1221.8)	(–819.4)	(–402.4)	(–1177.3)	(–789.2)	(–388.1)		
**$PrH_2C^{\bullet}$** [b]	–1631.6	–1228.3	–403.2	–1575.1	–1185.7	–389.4	19.3	–376.4
	(–1624.6)	(–1221.8)	(–402.7)	(–1565.6)	(–1177.3)	(–388.3)		
**$Me_2HC^{\bullet}$**	–1232.4	–838.6	–393.7	–1189.8	–809.0	–380.8	21.5	–368.5
	(–1225.0)	(–829.7)	(–395.3)	(–1180.5)	(–799.1)	(–381.4)		
**$EtMeHC^{\bullet}$** [b]	–1635.3	–1244.7	–390.6	–1579.0	–1201.0	–378.0	16.5	–366.8
	(–1627.5)	(–1234.3)	(–393.2)	(–1568.4)	(–1188.8)	(–379.7)		
**$PrMeHC^{\bullet}$**	–2038.5	–1647.7	–390.8	–1968.4	–1590.2	–378.2	[d]	[d]
	(–2030.1)	(–1636.7)	(–393.4)	(–1956.7)	(–1576.8)	(–379.9)		
**$Et_2HC^{\bullet}$**	–2038.3	–1650.7	–387.6	–1968.1	–1593.0	–375.1	[d]	[d]
	(–2029.9)	(–1638.9)	(–391.0)	(–1956.4)	(–1578.4)	(–378.0)		
**$Pr_2HC^{\bullet}$**	–2844.7	–2456.7	–388.0	–2747.0	–2371.4	–375.6	[d]	[d]
	(–2835.0)	(–2443.7)	(–391.3)	(–2732.8)	(–2354.6)	(–378.3)		
**$Me_3C^{\bullet}$** [b]	–1639.6	–1258.0	–381.6	–1583.4	–1213.5	–369.9	11.5	–361.6
	(–1631.0)	(–1244.5)	(–386.5)	(–1572.0)	(–1198.6)	(–373.4)		
**$EtMe_2C^{\bullet}$**	–2042.3	–1664.0	–378.3	–1972.1	–1605.5	–366.6	6.7	–359.7
	(–2033.1)	(–1649.1)	(–384.0)	(–1959.6)	(–1588.3)	(–371.3)		
**$PrMe_2C^{\bullet}$**	–2445.7	–2067.0	–378.7	–2361.7	–1994.7	–367.0	[d]	[d]
	(–2435.8)	(–2051.5)	(–384.3)	(–2347.9)	(–1976.3)	(–371.6)		
**$Et_3C^{\bullet}$**	–2848.1	–2476.1	–372.0	–2749.9	–2389.5	–360.4	[d]	[d]
	(–2837.6)	(–2458.3)	(–379.3)	(–2735.0)	(–2367.7)	(–367.4)		
**$Pr_3C^{\bullet}$**	–4058.4	–3685.0	–373.4	–3919.0	–3557.1	–361.9	[d]	[d]
	(–4046.6)	(–3665.5)	(–381.1)	(–3901.0)	(–3531.8)	(–369.2)		

[a] Computed at UBLYP-D3(BJ)/TZ2P (and at UM06-2X/TZ2P in parenthesis), enthalpies ΔH at 298.15 K and 1 atm. [b] The $C_4H_9^{\bullet}$ isomers are highlighted in gray. [c] Experimental heats of formation $\Delta H_f(R_3C^{\bullet})$ from ref. 24–31. [d] No experimental heats of formation ΔH_f available. [e] Radical enthalpy $\Delta H_{rad}(R_3C^{\bullet})$ estimated from the experimental heats of formation ΔH_f (eqn (6)). Heats of formation of gaseous $H^{\bullet}$ and $C^{\bullet\bullet}$ are 52.1 and 171.3 kcal mol^{-1}, respectively. Excitation of $C^{\bullet\bullet}$ to the $C^{\bullet\bullet\bullet\bullet}$ valence state is 96.5 kcal mol^{-1} (from ref. 24).

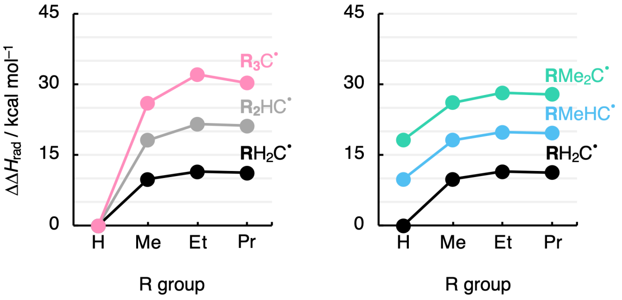

Figure S1. Substituent effect $\Delta\Delta H_{rad}$ as a function of the number and size of substituents $R^{\bullet}$ group in $R_3C^{\bullet}$ radicals ($R_3 = H_aMe_bEt_cPr_d$ with a+b+c+d = 3), computed at UM06-2X/TZ2P at 298.15 K and 1 atm.

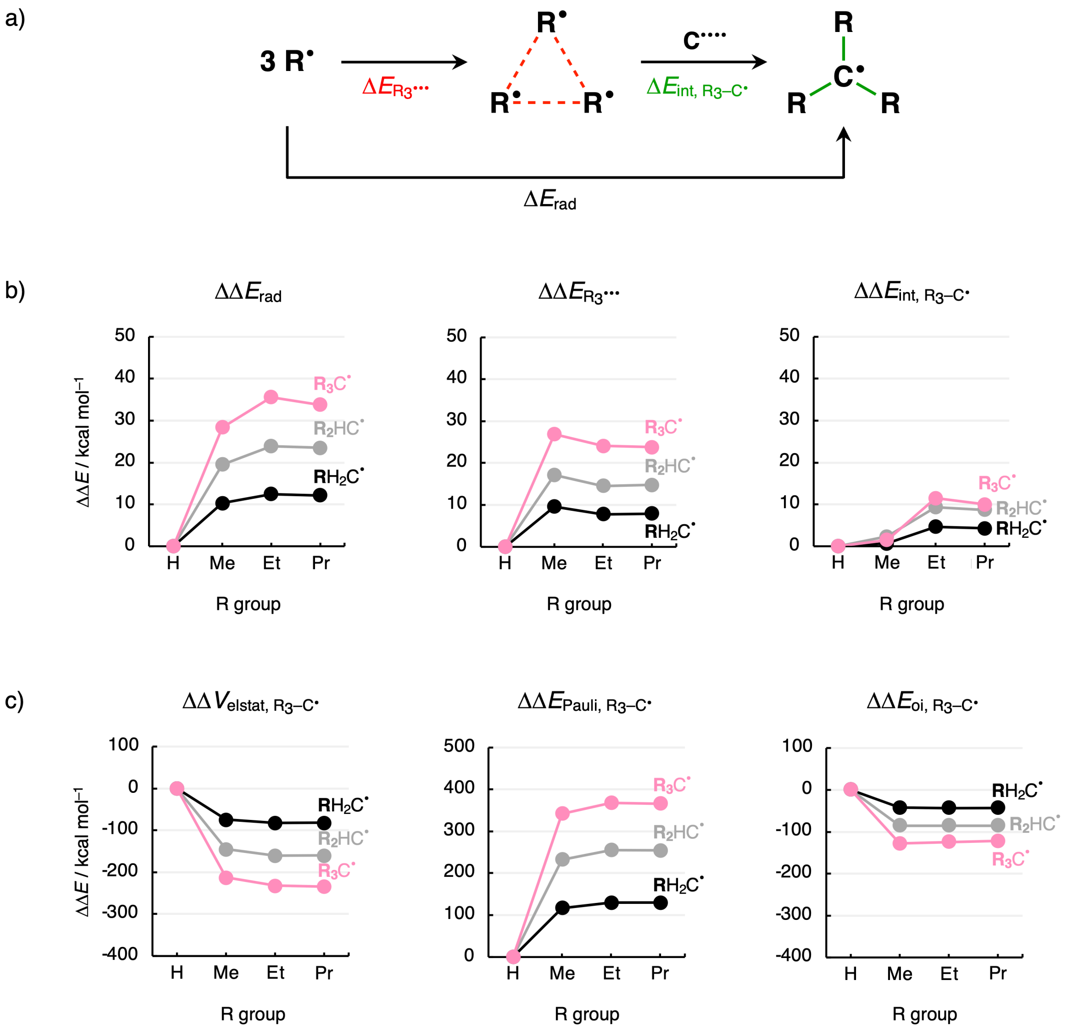

Figure S2. a) Analyzing $R_3C^{\bullet}$ in terms of two steps. b) Substituent effect $\Delta\Delta E_{rad}$ and its two components $\Delta\Delta E_{R_3\cdots}$ and $\Delta\Delta E_{int,R_3-C^{\bullet}}$ and c) energy decomposition analysis (EDA) of the substituent-effect component $\Delta E_{int,R_3-C^{\bullet}}$ as a function of the number and size of substituents $R^{\bullet}$ in $R_3C^{\bullet}$ radicals ($R_3 = H_aMe_bEt_cPr_d$ with a+b+c+d = 3), computed at UM06-2X/TZ2P.

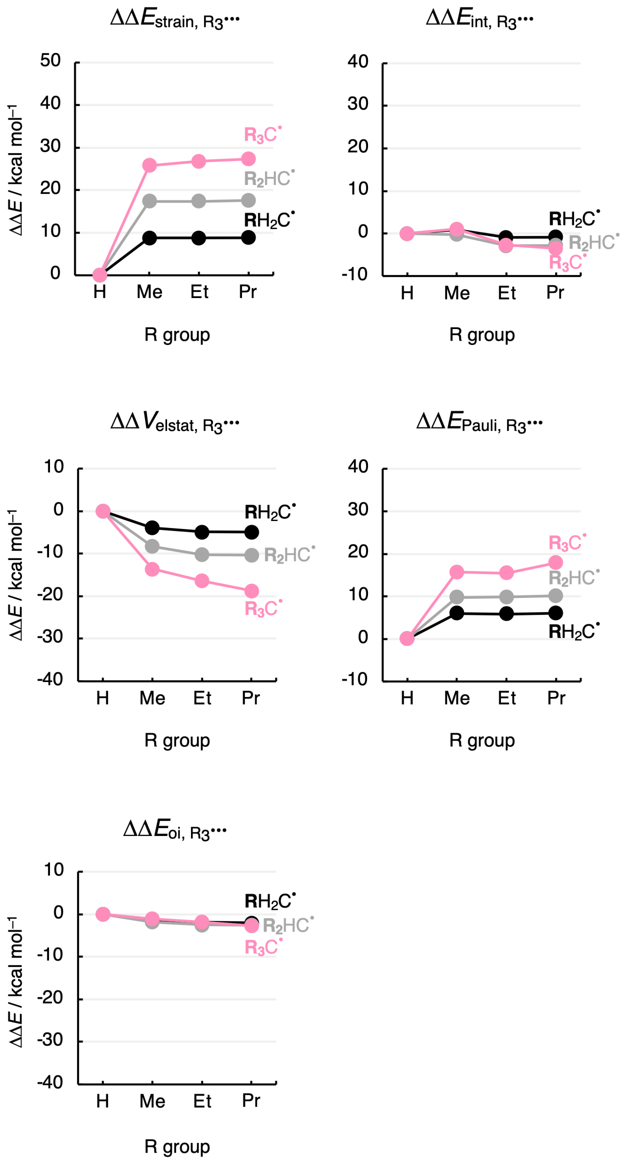

Figure S3. Activation strain model (ASM) and energy decomposition analysis (EDA) of the substituent energy $\Delta\Delta E_{R_3\cdots}$ as a function of the number and size of substituents R• in $R_3C^•$ radicals ($R_3 = H_aMe_bEt_cPr_d$ with a+b+c+d = 3), computed at UM06-2X/TZ2P.

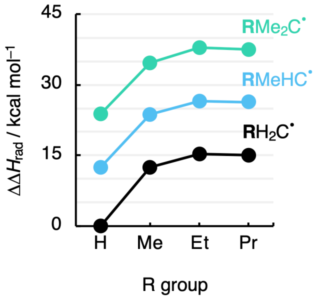

Figure S4. Substituent effect $\Delta\Delta H_{rad}$ as a function of the number and size of substituents R$^{\bullet}$ in $R_3C^{\bullet}$ radicals (R_3 = $H_aMe_bEt_cPr_d$ with a+b+c+d = 3), computed at UBLYP-D3(BJ)/TZ2P for 298.15 K and 1 atm.

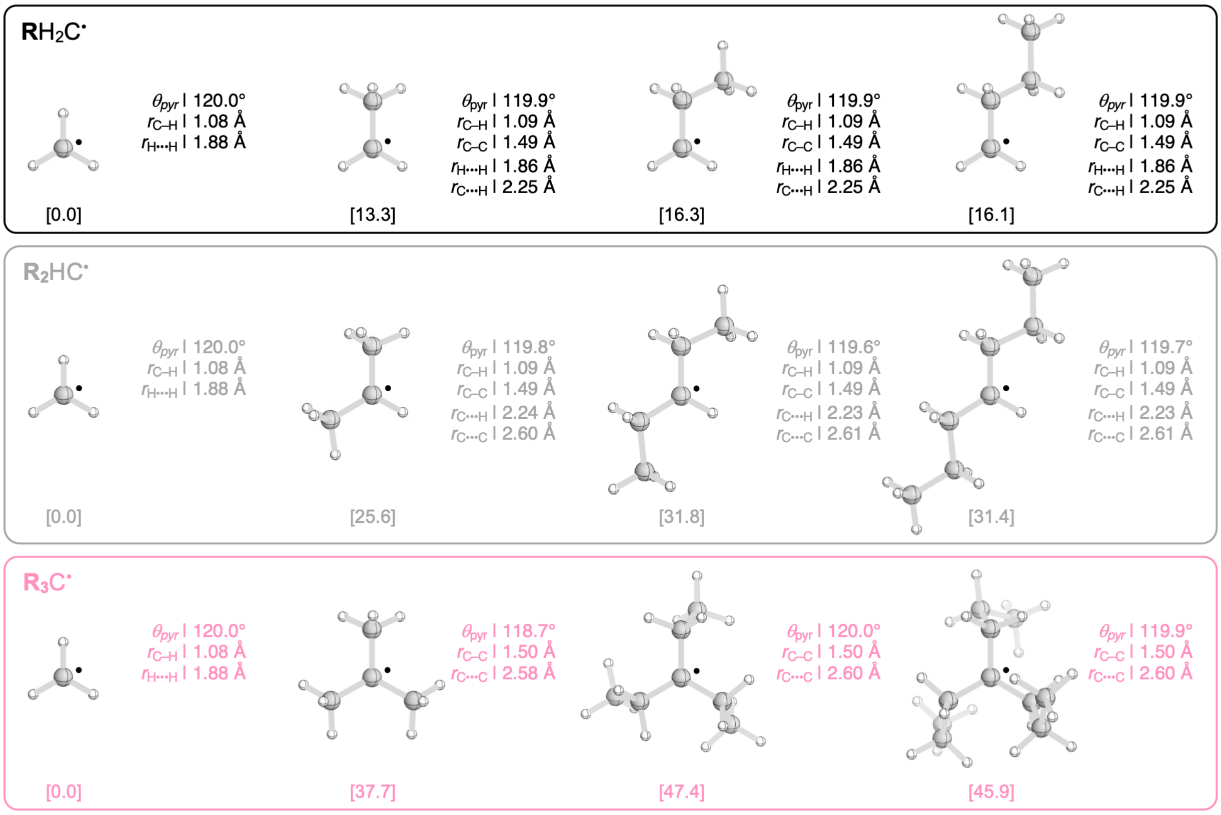

Figure S5. Equilibrium geometries of the $R_3C^{\bullet}$ radicals (R_3 = $H_aMe_bEt_cPr_d$ with a+b+c+d = 3) along with their relative radical energies $\Delta\Delta E_{rad}$ (in kcal mol^{-1}; in square brackets), computed at UBLYP-D3(BJ)/TZ2P. θ_{pyr} is the average of the three θ_{R-C-R} angles.

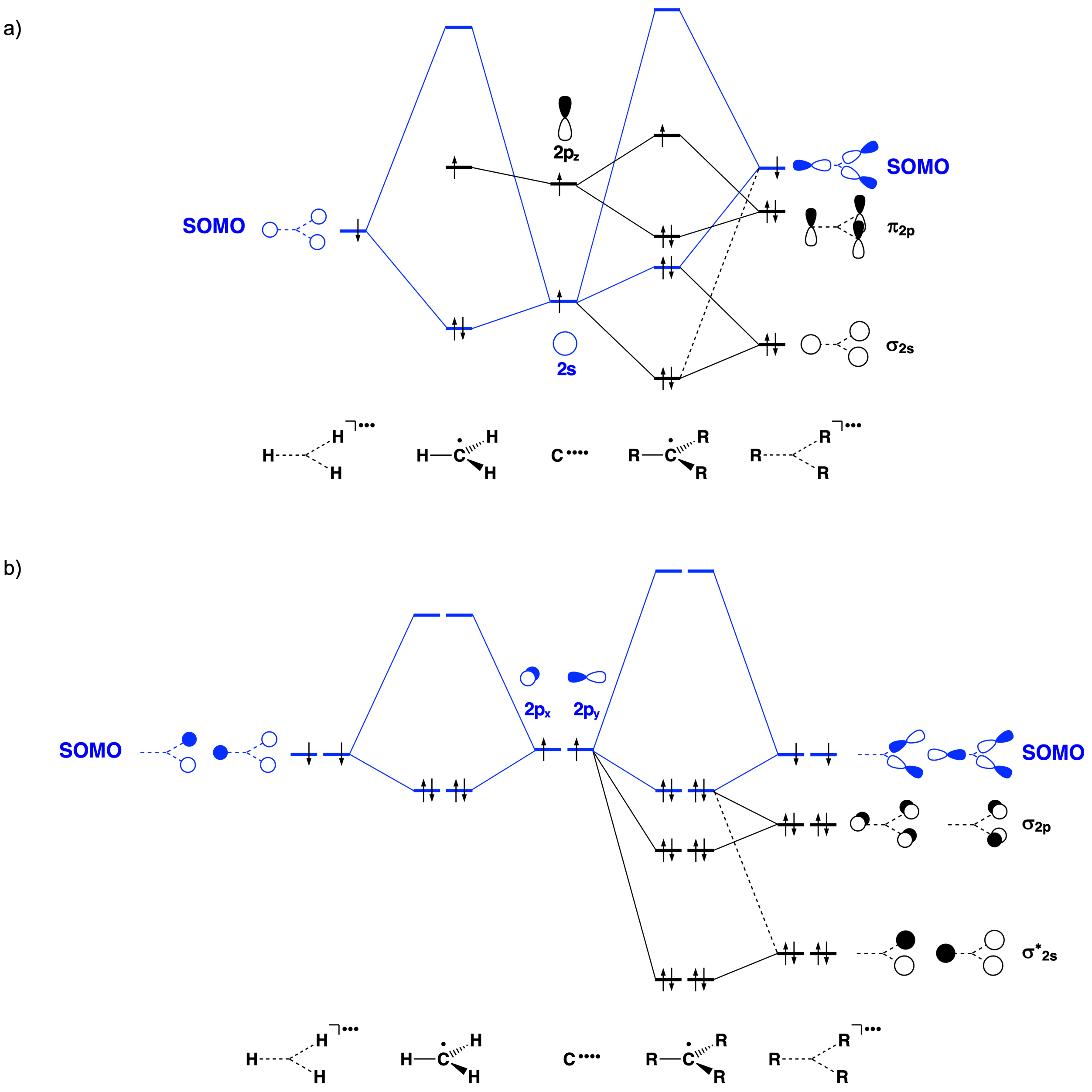

Figure S6. Schematic MO diagram of $R_3C^{\bullet}$ in terms of $R_3^{\bullet\bullet\bullet}$ interacting with $C^{\bullet\bullet\bullet\bullet}$ for R = H (left) and R = Me (right): a) in A_1 symmetry; b) in E_1 symmetry. Electron-pair bond (2c–2e$^-$ interaction) is shown in blue. For R = Et and Pr, the symmetry breaks down (see Figure S5), but the MOs are analogous to the ones depicted for R = Me.

Table S2. Orbital overlap S and orbital energy gap $\Delta\varepsilon$ (in eV) of the 2c–2e$^-$ interactions in $R_3C^•$ radicals (R_3 = $H_aMe_bEt_cPr_d$ with a+b+c+d = 3) in terms of $R_3^{•••}$ interacting with $C^{••••}$.[a]

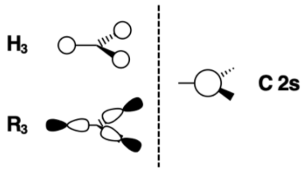

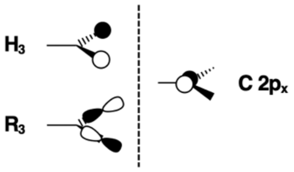

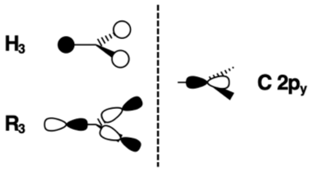

	1: SOMO–SOMO		2: SOMO–SOMO		3: SOMO–SOMO	
**System**	S[b]	$\Delta\varepsilon$	S[b]	$\Delta\varepsilon$	S[b]	$\Delta\varepsilon$
**H_3–$C^•$**	0.79	12.0	0.63	5.8	0.63	5.8
	0.84	*1.7*	*0.79*	*5.4*	*0.79*	*5.4*
**Me_3–$C^•$**	0.66	9.6	0.47	4.7	0.47	4.7
	0.62	*0.3*	*0.40*	*3.7*	*0.40*	*3.7*
**Et_3–$C^•$**	0.63	10.3	0.48	5.2	0.48	5.2
	0.55	*1.4*	*0.40*	*3.0*	*0.40*	*3.0*
**Pr_3–$C^•$**	0.63	10.3	0.47	5.2	0.47	5.1
	0.53	*1.5*	*0.40*	*3.0*	*0.40*	*3.0*

[a] Computed at UBLYP-D3(BJ)/TZ2P; see Figure S6 for the schematic MO diagram. [b] Orbital overlap S and orbital energy gap $\Delta\varepsilon$ between alpha-spin and beta-spin orbitals in plain text and italics, respectively.

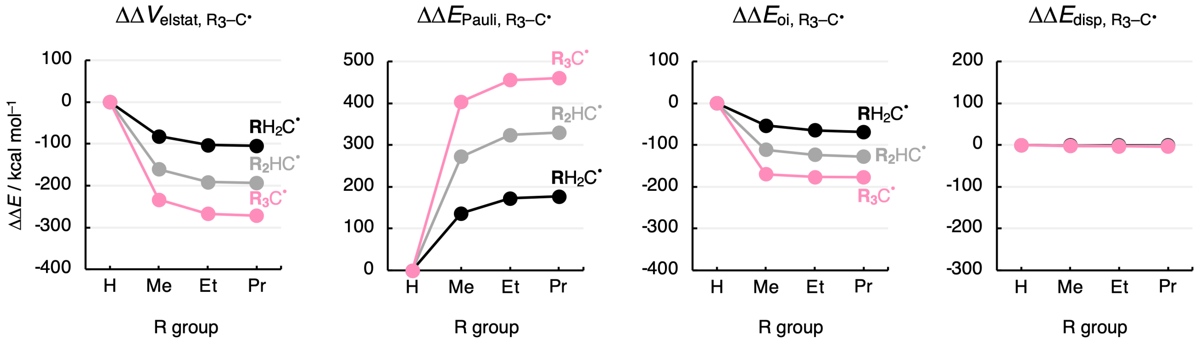

Figure S7. Energy decomposition analysis (EDA) of the substituent-effect component $\Delta E_{\mathrm{int},R_3-C^•}$ as a function of the number and size of substituents $R^•$ in $R_3C^•$ radicals (R_3 = $H_aMe_bEt_cPr_d$ with a+b+c+d = 3), computed at UBLYP-D3(BJ)/TZ2P.

a)

$C^{\bullet\bullet\bullet\bullet}$	$H_3^{\bullet\bullet\bullet}$ 1A'₁	1E':1	1E':2
2p_z	0.00<br>*0.00*	0.00<br>*0.00*	0.00<br>*0.00*
2p_y	0.00<br>*0.00*	0.00<br>*0.00*	0.63<br>*0.79*
2p_x	0.00<br>*0.00*	0.63<br>*0.79*	0.00<br>*0.00*
2*s*	0.79<br>*0.84*	0.00<br>*0.00*	0.00<br>*0.00*
1*s*	0.12<br>*0.12*	0.00<br>*0.00*	0.00<br>*0.00*

b)

$C^{\bullet\bullet\bullet\bullet}$	$H_2Me^{\bullet\bullet\bullet}$ 1A'	2A'	1A"	3A'	4A'	2A"	5A'
2p_z	0.00<br>*0.00*	0.00<br>*0.00*	0.00<br>*0.00*	0.21<br>*0.28*	0.02<br>*0.03*	0.00<br>*0.00*	0.02<br>*0.02*
2p_y	0.07<br>*0.09*	0.33<br>*0.44*	0.00<br>*0.00*	0.00<br>*0.00*	0.05<br>*0.25*	0.00<br>*0.00*	0.53<br>*0.56*
2p_x	0.00<br>*0.00*	0.00<br>*0.00*	0.23<br>*0.35*	0.00<br>*0.00*	0.00<br>*0.00*	0.64<br>*0.72*	0.00<br>*0.00*
2*s*	0.03<br>*0.04*	0.26<br>*0.33*	0.00<br>*0.00*	0.00<br>*0.00*	0.73<br>*0.76*	0.00<br>*0.00*	0.19<br>*0.03*
1*s*	0.00<br>*0.00*	0.02<br>*0.03*	0.00<br>*0.00*	0.00<br>*0.00*	0.12<br>*0.12*	0.00<br>*0.00*	0.02<br>*0.02*

c)

$C^{\bullet\bullet\bullet\bullet}$	$H_2Et^{\bullet\bullet\bullet}$ 1A	2A	3A	4A	5A	6A	7A	8A	9A	10A	11A
2p_z	0.00<br>*0.00*	0.00<br>*0.00*	0.02<br>*0.03*	0.03<br>*0.05*	0.16<br>*0.22*	0.04<br>*0.06*	0.04<br>*0.06*	0.13<br>*0.15*	0.02<br>*0.00*	0.00<br>*0.02*	0.02<br>*0.02*
2p_y	0.02<br>*0.02*	0.07<br>*0.09*	0.25<br>*0.37*	0.22<br>*0.23*	0.00<br>*0.00*	0.09<br>*0.01*	0.03<br>*0.09*	0.00<br>*0.02*	0.02<br>*0.31*	0.11<br>*0.03*	0.55<br>*0.53*
2p_x	0.00<br>*0.01*	0.00<br>*0.00*	0.06<br>*0.08*	0.09<br>*0.15*	0.07<br>*0.12*	0.12<br>*0.17*	0.14<br>*0.20*	0.04<br>*0.06*	0.00<br>*0.04*	0.61<br>*0.73*	0.10<br>*0.05*
2*s*	0.00<br>*0.00*	0.04<br>*0.04*	0.18<br>*0.24*	0.19<br>*0.23*	0.00<br>*0.04*	0.16<br>*0.34*	0.07<br>*0.35*	0.00<br>*0.06*	0.73<br>*0.58*	0.03<br>*0.00*	0.13<br>*0.08*
1*s*	0.00<br>*0.00*	0.00<br>*0.00*	0.01<br>*0.02*	0.02<br>*0.02*	0.00<br>*0.00*	0.02<br>*0.05*	0.00<br>*0.05*	0.00<br>*0.00*	0.12<br>*0.09*	0.00<br>*0.00*	0.00<br>*0.03*

d)

$C^{\bullet\bullet\bullet\bullet}$	$H_2Pr^{\bullet\bullet\bullet}$ 1A	2A	3A	4A	5A	6A	7A	8A	9A	10A	11A	12A	13A	14A	15A
2p_z	0.00<br>*0.00*	0.00<br>*0.00*	0.00<br>*0.00*	0.02<br>*0.04*	0.00<br>*0.00*	0.05<br>*0.08*	0.11<br>*0.16*	0.02<br>*0.01*	0.05<br>*0.06*	0.14<br>*0.18*	0.04<br>*0.03*	0.08<br>*0.08*	0.02<br>*0.06*	0.01<br>*0.00*	0.02<br>*0.02*
2p_y	0.01<br>*0.02*	0.00<br>*0.00*	0.07<br>*0.09*	0.18<br>*0.29*	0.23<br>*0.29*	0.14<br>*0.13*	0.00<br>*0.00*	0.08<br>*0.03*	0.07<br>*0.05*	0.00<br>*0.00*	0.00<br>*0.14*	0.00<br>*0.02*	0.02<br>*0.30*	0.10<br>*0.03*	0.54<br>*0.53*
2p_x	0.00<br>*0.01*	0.00<br>*0.00*	0.00<br>*0.00*	0.05<br>*0.08*	0.02<br>*0.00*	0.11<br>*0.12*	0.06<br>*0.10*	0.05<br>*0.03*	0.12<br>*0.20*	0.08<br>*0.10*	0.10<br>*0.10*	0.03<br>*0.03*	0.00<br>*0.11*	0.62<br>*0.73*	0.10<br>*0.04*
2*s*	0.00<br>*0.00*	0.00<br>*0.00*	0.04<br>*0.04*	0.12<br>*0.17*	0.19<br>*0.25*	0.12<br>*0.13*	0.00<br>*0.01*	0.11<br>*0.31*	0.14<br>*0.33*	0.00<br>*0.00*	0.03<br>*0.19*	0.00<br>*0.05*	0.73<br>*0.58*	0.03<br>*0.00*	0.13<br>*0.07*
1*s*	0.00<br>*0.00*	0.00<br>*0.00*	0.00<br>*0.00*	0.00<br>*0.01*	0.02<br>*0.02*	0.01<br>*0.01*	0.00<br>*0.00*	0.01<br>*0.04*	0.02<br>*0.05*	0.00<br>*0.00*	0.00<br>*0.03*	0.00<br>*0.00*	0.12<br>*0.09*	0.00<br>*0.00*	0.00<br>*0.03*

Figure S8. Orbital overlap integrals in $R_3C^{\bullet}$ radicals ($R_3 = H_aMe_bEt_cPr_d$ with a+b+c+d = 3) of $R_3^{\bullet\bullet\bullet}$ interacting with $C^{\bullet\bullet\bullet\bullet}$ for a) $H_3C^{\bullet}$ in D_{3h} symmetry, b) $H_2MeC^{\bullet}$ in C_S symmetry, c) $H_2EtC^{\bullet}$ in C_1 symmetry, and d) $H_2PrC^{\bullet}$ in C_1 symmetry. Occupied–occupied overlap in red, and electron-pair bond overlap in green. Orbital overlap between alpha-spin and beta-spin orbitals in plain text and italics, respectively. Computed at UBLYP-D3(BJ)/TZ2P.

Supporting Discussion 1 | Pauli repulsion

As can be seen from Figure 3c, increasing the number of substituents R from $RH_2C^•$ to $R_2HC^•$ to $R_3C^•$ results in a larger increase in Pauli repulsion than increasing the substituent size from R = Me to Et to Pr. The number of closed-shell orbitals, absent in $H_3^{•••}$, increases going from $H_3C^•$ to $H_2MeC^•$ (compare Figures 8a and 8b), thus providing an additional destabilizing contribution to $\Delta\Delta E_{\text{Pauli,R3–C•}}$. This effect is nearly additive going from $H_3C^•$ to $H_2MeC^•$ to $HMe_2C^•$ to $Me_3C^•$ (see Figure 3c). However, as R in $H_2RC^•$ increases in size from Me to Et to Pr, the increase in $\Delta\Delta E_{\text{Pauli,R3–C•}}$ is less pronounced (see Figure 3c) despite the increasing number of closed-shell orbitals along this series (compare Figures 8b and 8d). This is so because although the number of occupied–occupied overlap increases, their magnitude decreases because the occupied orbitals are more delocalized over the longer R group and, therefore, each of them has a lower amplitude at the contact point close to the carbon center. That is the reason why the increase in $\Delta\Delta E_{\text{Pauli,R3–C•}}$ becomes gradually less steep going from H to Me to Et to Pr.

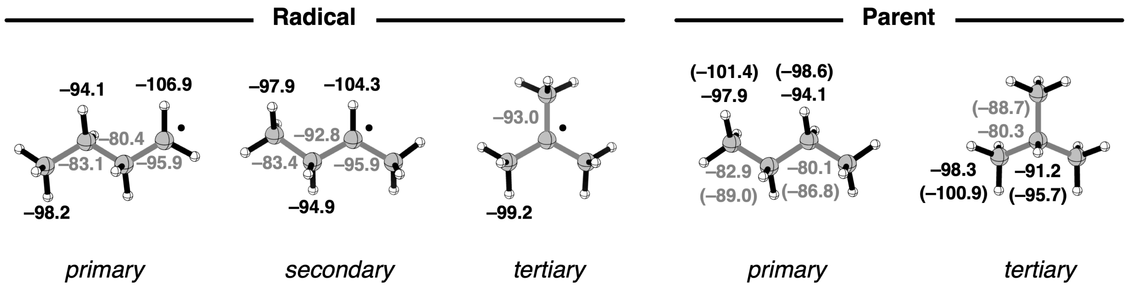

Figure S9. C–H (in black) and C–C (in gray) bond enthalpies (ΔH_{bond}(C–X), in kcal mol^{-1}) of the $C_4H_9^{•}$ radical isomers and the corresponding C_4H_{10} parent molecules computed at (U)BLYP-D3(BJ)/TZ2P. Bond enthalpies in parentheses are calculated from experimental heats of formation ΔH_f taken from ref. 24–31.

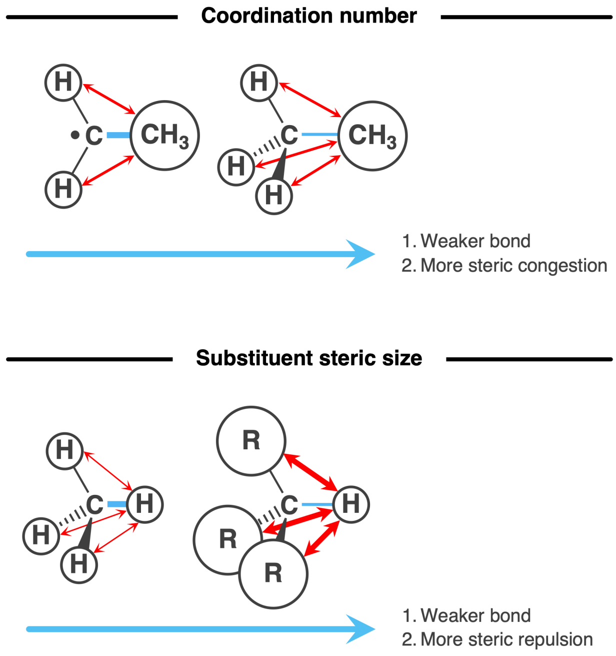

Figure S10. Schematic representation of the effect of the coordination number and the effect of the steric size of the substituents on the strength of C–H and C–C bonds.

Supporting Discussion 2 | Strength of C–H and C–C bonds

The reason why the C–H bond is more sensitive to the steric size of the groups R than the C–C bond is the lack of stabilizing interactions to counteract the increase in steric Pauli repulsion (see Table S3 and Figure S9). The increase in ΔE_{Pauli} going from $H_3C–CH_3$ to $Me_3C–CH_3$ ($\Delta\Delta E_{Pauli}$ = 35.5 kcal mol^{-1}) is larger than from $H_3C–H$ to $Me_3C–H$ ($\Delta\Delta E_{Pauli}$ = 27.1 kcal mol^{-1}) because of the introduction of closed-shell subvalence orbitals in $^{•}CH_3$ compared to $^{•}H$, which provides additional destabilizing contribution to ΔE_{Pauli}. The C–C bond experiences more steric repulsion than the C–H bond, but, at the same time, it also benefits from stabilizing interactions that favor steric crowding. For example, the electrostatic interactions ΔV_{elstat} and dispersion interactions ΔE_{disp} energy terms become much more stabilizing from $H_3C–CH_3$ to $Me_3C–CH_3$ ($\Delta\Delta V_{elstat}$ = –20.6 kcal mol^{-1} and $\Delta\Delta E_{disp}$ = –2.9 kcal mol^{-1}) than from $H_3C–H$ to $Me_3C–H$ ($\Delta\Delta V_{elstat}$ = –12.9 kcal mol^{-1} and $\Delta\Delta E_{disp}$ = –0.5 kcal mol^{-1}). Therefore, stabilizing interactions somewhat counteract the increase in steric repulsion due to the introduction of bulky substituents, causing the C–C bond to be less destabilized than it would be solely based on steric repulsion arguments.

Table S3. $Me_mH_{3-m}C–X$ (m = 0–3; X = H, CH_3) bond enthalpies and energies (ΔH and ΔE), activation strain model and energy decomposition analysis terms (in kcal mol^{-1}), and C–X bond lengths (in Å).[a]

Systems	m	ΔH	ΔE	ΔE_{strain}	ΔE_{int}	ΔV_{elstat}	ΔE_{Pauli}	ΔE_{oi}	ΔE_{disp}	$d_{C–X}$
**$H_3C–H$**	0	–102.7	–110.2	7.2	–117.4	–58.0	73.4	–132.4	–0.4	1.094
**$MeH_2C–H$**	1	–97.7	–105.3	7.2	–112.6	–63.2	84.9	–133.6	–0.6	1.097
**$Me_2HC–H$**	2	–93.9	–101.6	7.2	–108.8	–67.5	93.5	–134.0	–0.8	1.099
**$Me_3C–H$**	3	–91.2	–98.6	6.9	–105.5	–70.9	100.5	–134.2	–0.9	1.101
**$H_3C–CH_3$**	0	–85.2	–92.1	18.4	–110.4	–124.2	184.4	–168.6	–2.0	1.538
**$MeH_2C–CH_3$**	1	–82.7	–89.2	18.3	–107.4	–133.9	201.4	–172.0	–2.9	1.538
**$Me_2HC–CH_3$**	2	–80.3	–86.6	18.4	–104.9	–140.7	212.7	–173.0	–3.9	1.541
**$Me_3C–CH_3$**	3	–78.0	–84.0	18.3	–102.3	–144.8	219.9	–172.5	–4.9	1.545
**$H_2C^{•}–H$**	0	–111.0	–117.3	2.1	–119.5	–54.9	63.7	–127.8	–0.4	1.084
**$MeHC^{•}–H$**	1	–106.8	–113.1	2.3	–115.5	–59.8	73.2	–128.2	–0.7	1.086
**$Me_2C^{•}–H$**	2	–103.9	–110.0	2.5	–112.6	–63.0	79.3	–128.0	–0.9	1.088
**$H_2C^{•}–CH_3$**	0	–98.5	–104.1	12.9	–116.9	–131.3	191.4	–175.1	–1.9	1.490
**$MeHC^{•}–CH_3$**	1	–95.6	–100.8	13.1	–113.9	–141.1	207.2	–177.3	–2.8	1.493
**$Me_2C^{•}–CH_3$**	2	–93.0	–98.0	13.8	–111.8	–146.9	216.5	–177.7	–3.7	1.498

[a] Computed at (U)BLYP-D3(BJ)/TZ2P, enthalpies ΔH at 298.15 K and 1 atm.

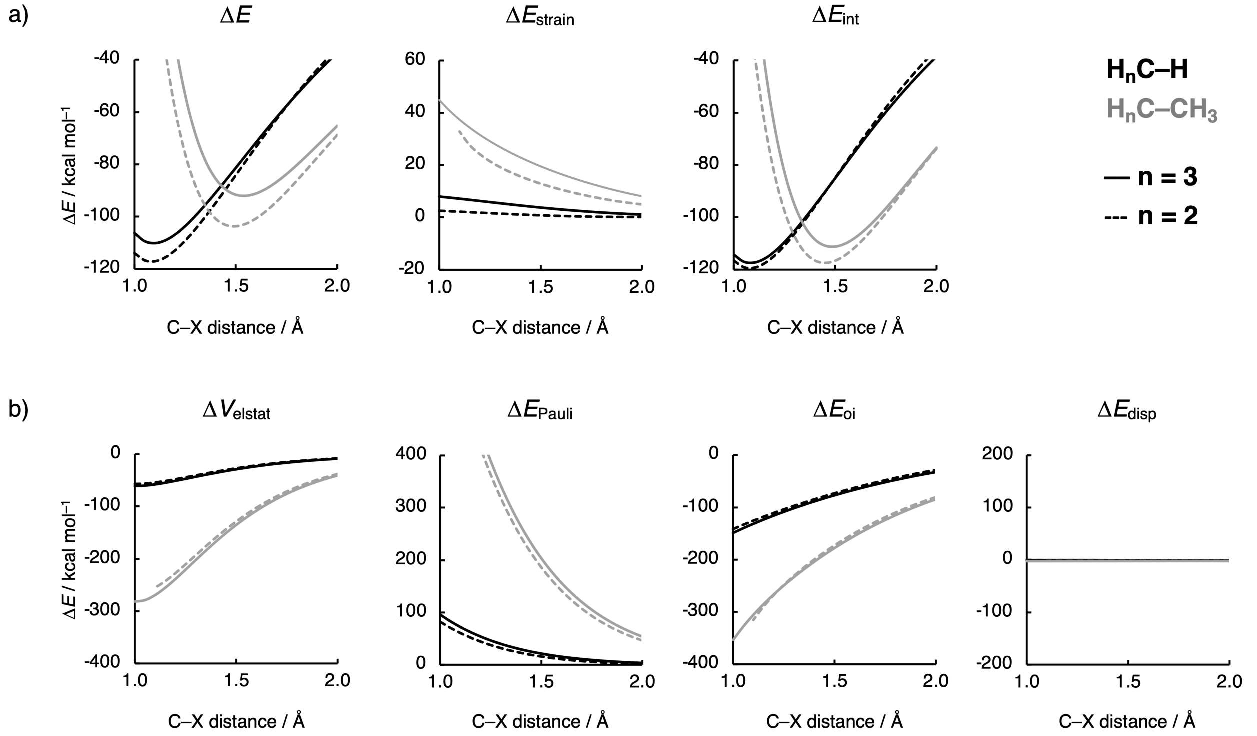

Figure S11. a) Activation strain model and b) energy decomposition analysis as a function of the C–X distance of the homolytic H_nC–X bond dissociation (n = 3, 2; X = H, CH_3). Computed at (U)BLYP-D3(BJ)/TZ2P.

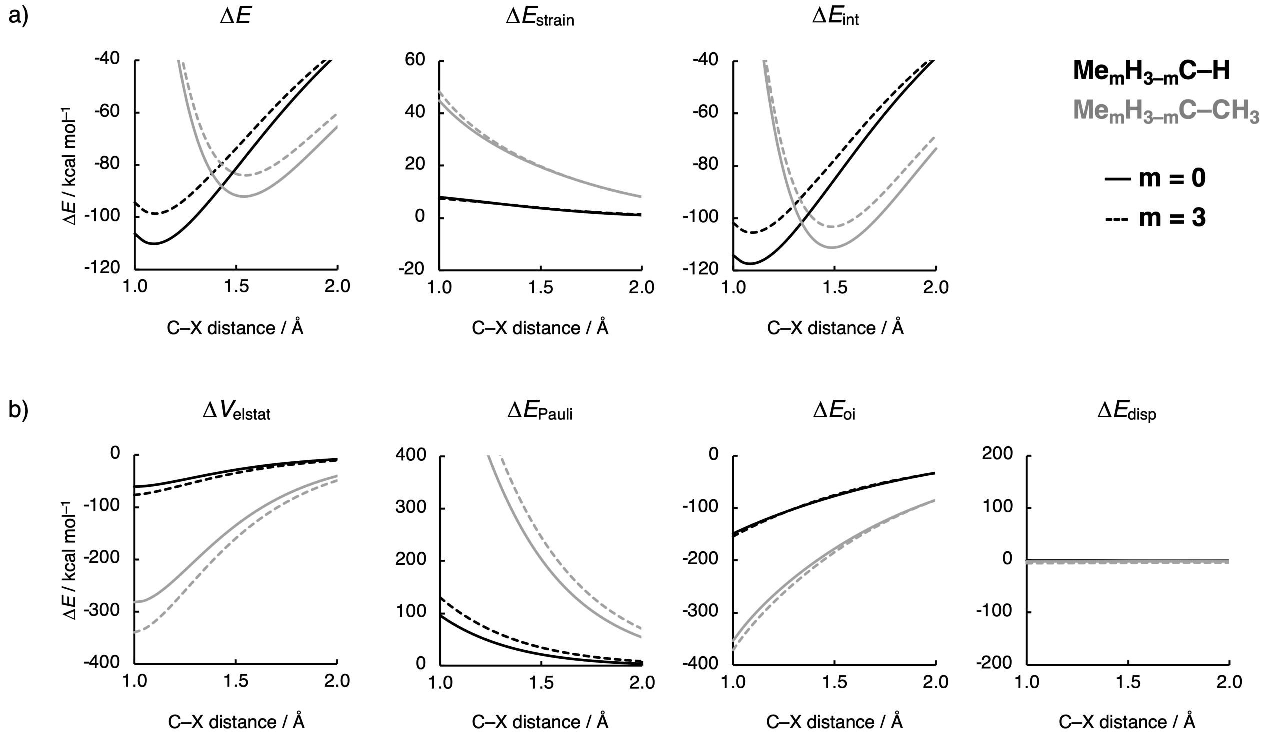

Figure S12. a) Activation strain model and b) energy decomposition analysis as a function of the C–X distance of the homolytic $Me_mH_{3-m}C$–X bond dissociation (m = 0, 3; X = H, CH_3). Computed at (U)BLYP-D3(BJ)/TZ2P.

Supporting Discussion 3 | Heat of formation of the $R_3C^{\bullet}$ radicals

The radical isomers can be gradually constructed from their shared building blocks to show how the variation in C–H and C–C bond strength explains the differences in the heats of formation. We start from the atoms in their valence configuration (*i.e.*, $C^{\bullet\bullet\bullet\bullet}$ and $H^{\bullet}$) and then form the carbon backbone common to all radical isomers (*i.e.*, H_3C–$C^{\bullet\bullet}$–$H_2C^{\bullet}$) together with an $H_2C^{\bullet\bullet}$ group and two $H^{\bullet}$ atoms (Figure S13). The energy change associated with this step is the same for all isomers because this step leads to the same intermediate set of species. From this point, two distinct paths can be followed. On the left side of Figure S13, one $H^{\bullet}$ atom is attached to the middle –$C^{\bullet\bullet}$– carbon atom and the $H_2C^{\bullet\bullet}$ group to the right end of the carbon backbone (*i.e.*, –$H_2C^{\bullet}$), thereby forming the H_3C–$CH^{\bullet}$–H_2C–$H_2C^{\bullet}$ carbon chain. This path leads to either the primary or the secondary radical, depending on where the final $H^{\bullet}$ atom is added, which is the stability-determining step in this case. Therefore, the reason the secondary radical is more stable than the primary radical is that the C–H bond in a primary (*i.e.*, less sterically hindered) carbon is stronger than in a secondary carbon (Figure S9 and Supporting Discussion 2).

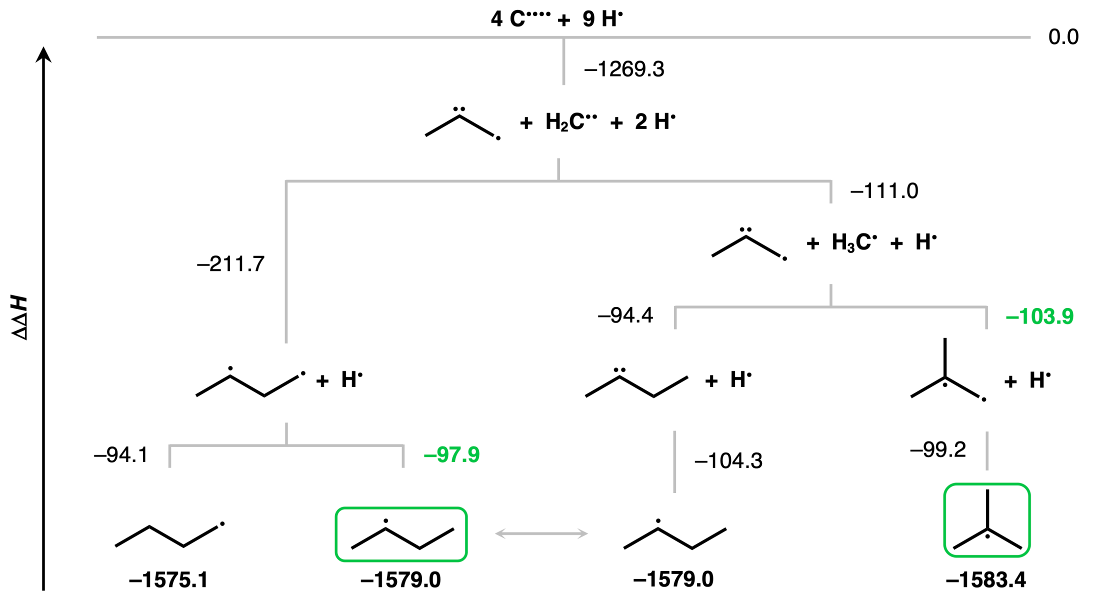

Figure S13. Bond enthalpy (in kcal mol^{-1}) of the step-by-step formation of the $C_4H_9^{\bullet}$ isomers relative to the atoms in their valence configuration (*i.e.*, $C^{\bullet\bullet\bullet\bullet}$ and $H^{\bullet}$). The relative stability determining step is highlighted in green. Computed at (U)BLYP-D3(BJ)/TZ2P.

On the second path, on the right side of Figure S13, we have the H_3C–$C^{\bullet\bullet}$–$H_2C^{\bullet}$ carbon backbone with a $H_3C^{\bullet}$ group and an $H^{\bullet}$ atom. We can now form either secondary or tertiary isomers depending on how the three building blocks are put together. If the $H_3C^{\bullet}$ group is attached to the right end of the carbon backbone (*i.e.*, –$H_2C^{\bullet}$) followed by the insertion of the $H^{\bullet}$ atom at the middle –$C^{\bullet\bullet}$– carbon, the secondary isomer is formed. In the opposite order, if the $H_3C^{\bullet}$ group is added at

the middle $-C^{••}-$ carbon, followed by the $H^{•}$ atom at the right $-H_2C^{•}$ carbon, the tertiary isomer is obtained. The step that determines the higher stability of the tertiary isomer over the secondary isomer is the insertion of the $H_3C^{•}$ group ($\Delta\Delta H$ = –94.4 and –103.9 kcal mol^{-1}, respectively). The C–C bond strength is more sensitive to the coordination number around the pertinent carbon atom than the C–H bond (Table S3 and Figure S11) and, therefore, the isomer that has C–C bonds to low coordination carbons, as in the tertiary $C_4H_9^{•}$ radical, is more stable.

Supporting Discussion 4 | Heat of formation of the R_3C–H parent molecules

Similar to the $C_4H_9^•$ alkyl radical isomers, the C_4H_{10} alkane isomers become more stable going from primary to tertiary (*i.e.*, from linear to branched) alkanes (ΔH_f = –30.0 kcal mol^{-1} and –32.1 kcal mol^{-1}, respectively). Again, this is so despite the *destabilizing* effect of the substituents on the carbon center as contained in $\Delta H_{par}(R_3C–H)$. The branched isomer is more stable than the linear one because the substituents R themselves, $-\Delta H_{atomization}(R^•)$, become more stable due to the stronger C–H bonds therein (Figure S14 and Table S4).

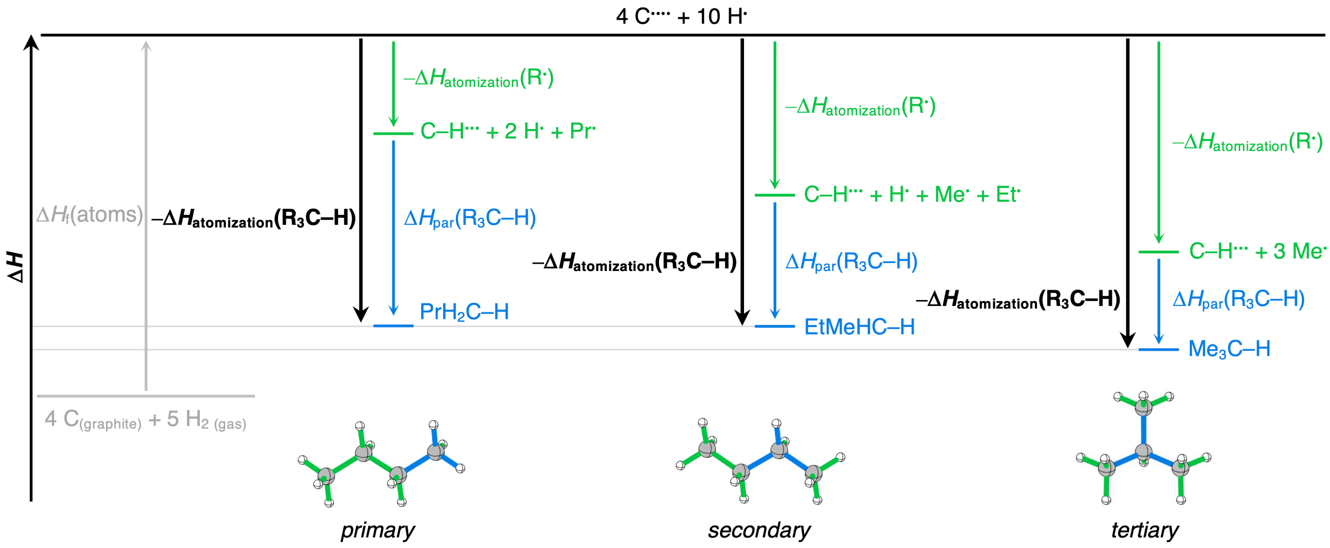

Figure S14. Thermochemical cycle of formation (in kcal mol^{-1}) of the C_4H_{10} isomers of the R_3C–H parent molecules ($R_3 = H_aMe_bEt_cPr_d$ with a+b+c+d = 3).

The reason why more branched alkanes have a more stable heat of formation than linear ones, despite having the same overall number of C–H and C–C bonds, is that the C–H bonds in the former are stronger than in the latter (Figure S9). This can be alternatively shown by gradually constructing each alkane isomer from the building blocks they have in common and analyzing the associated enthalpy energy change (Figure S15). Similar to the thermochemical cycle in Figure S14, we start from the atoms in their valence configuration (*i.e.*, $C^{••••}$ and $H^•$). Then, we form the carbon backbone shared by both isomers (*i.e.*, $H_3C–HC^•–H_2C^•$) plus a $H_3C^•$ group and a $H^•$ atom. The energy change associated with this step is the same for both isomers because this step leads to the same set of intermediate species. From this point, we can form either isomer depending on how the three building blocks are put together. If the $H_3C^•$ group is attached to the right end of the carbon backbone, at the primary $–H_2C^•$ carbon, followed by the addition of the $H^•$ atom at the middle, secondary $–HC^•–$ carbon, the linear C_4H_{10} isomer is formed. In the opposite order, that is, the $H_3C^•$ group at the secondary $–HC^•–$ carbon followed by the $H^•$ atom at the primary $–H_2C^•$ carbon, the branched C_4H_{10} isomer is obtained. The step that determines the higher stability of the branched

isomer over the linear isomer is the H• atom addition (ΔΔH = –94.1 and –98.3 kcal mol^{-1}, respectively). As discussed in Supporting Discussion 2, the C–H bond is more sensitive to the steric size of the groups R than the C–C and, therefore, the isomer that possesses C–H bonds to less sterically hindered carbon atoms (*i.e.*, primary carbons), that is, more branched alkanes, is more stable.

Table S4. Electronic energies and enthalpies (in kcal mol^{-1}) of the R_3C–H parent molecules (R_3 = $H_aMe_bEt_cPr_d$ with a+b+c+d = 3) according to the thermochemical cycle in Figure S14.[a,b]

Systems [b]	–ΔE_{atom} (R_3C–H)	–ΔE_{atom} (R•)	ΔE_{par} (R_3C–H)	–ΔH_{atom} (R_3C–H)	–ΔH_{atom} (R•)	ΔH_{par} (R_3C–H)	ΔH_f (R_3C–H)[c]	ΔH_{par} (R_3C–H)[e]
**H_3C–H**	–529.5 (–526.5)	–174.2 (–174.9)	–355.3 (–351.6)	–507.2 (–503.1)	–170.9 (–171.2)	–336.3 (–331.9)	–17.9	–333.3
**MeH_2C–H**	–930.7 (–927.1)	–593.5 (–589.7)	–337.2 (–337.4)	–894.2 (–889.1)	–575.4 (–570.8)	–318.8 (–318.3)	–20.2	–318.6
**EtH_2C–H**	–1333.9 (–1329.7)	–999.6 (–994.3)	–334.3 (–335.4)	–1283.7 (–1277.4)	–967.4 (–960.5)	–316.3 (–316.9)	–24.8	–316.5
**PrH_2C–H** [b]	–1737.1 (–1732.3)	–1402.6 (–1396.7)	–334.5 (–335.6)	–1673.1 (–1665.5)	–1356.6 (–1348.5)	–316.5 (–317.0)	–30.0	–317.2
**Me_2HC–H**	–1333.9 (–1329.7)	–1012.9 (–1004.5)	–321.0 (–325.2)	–1283.7 (–1277.3)	–979.9 (–970.3)	–303.8 (–307.0)	–24.8	–306.2
**EtMeHC–H** [b]	–1737.1 (–1732.3)	–1418.9 (–1409.1)	–318.2 (–323.2)	–1673.1 (–1665.6)	–1371.9 (–1360.0)	–301.2 (–305.6)	–30.0	–304.8
**PrMeHC–H**	–2140.2 (–2134.8)	–1821.9 (–1811.5)	–318.3 (–323.3)	–2062.5 (–2053.8)	–1761.1 (–1748.1)	–301.4 (–305.7)	–35.0	–305.2
**Et_2HC–H**	–2140.2 (–2134.8)	–1824.9 (–1813.7)	–315.3 (–321.1)	–2062.5 (–2053.7)	–1763.9 (–1749.7)	–298.5 (–304.0)	–35.0	–303.0
**Pr_2HC–H**	–2946.5 (–2939.9)	–2630.9 (–2618.5)	–315.6 (–321.4)	–2841.2 (–2830.1)	–2542.3 (–2525.8)	–298.9 (–304.3)	–44.9	–303.9
**Me_3C–H** [b]	–1738.2 (–1733.7)	–1432.2 (–1419.4)	–306.0 (–314.3)	–1674.5 (–1667.3)	–1384.4 (–1369.9)	–290.2 (–297.4)	–32.1	–296.5
**$EtMe_2C$–H**	–2140.8 (–2135.8)	–1838.2 (–1824.0)	–302.6 (–311.8)	–2063.3 (–2054.9)	–1776.4 (–1759.5)	–286.9 (–295.4)	–36.9	–294.7
**$PrMe_2C$–H**	–2544.0 (–2538.2)	–2241.2 (–2226.4)	–302.8 (–311.8)	–2452.7 (–2443.0)	–2165.6 (–2147.6)	–287.1 (–295.4)	–41.7	–294.9
**Et_3C–H**	–2945.7 (–2939.7)	–2650.3 (–2633.1)	–295.4 (–306.6)	–2840.4 (–2829.9)	–2560.4 (–2538.9)	–280.0 (–291.0)	–45.3	–289.7
**Pr_3C–H**	–4155.4 (–4147.2)	–3859.2 (–3840.4)	–296.2 (–306.8)	–4008.8 (–3994.3)	–3728.0 (-3703.1)	–280.8 (-291.3)	[d]	[d]

[a] Computed at (U)BLYP-D3(BJ)/TZ2P (and at (U)M06-2X/TZ2P in parenthesis), enthalpies ΔH at 298.15 K and 1 atm. [b] The C_4H_{10} isomers are highlighted in gray. [c] Experimental heats of formation ΔH_f(R_3C–H) from ref. 28,29. [d] No experimental heats of formation ΔH_f available. [e] Parent enthalpy ΔH_{par}(R_3C–H) estimated from the experimental heats of formation ΔH_f. Heats of formation of gaseous H• and CH• are 52.1 and 142.0 kcal mol^{-1}, respectively. Excitation from the ground state CH• to the first excited state CH••• is 17.1 kcal mol^{-1} (from ref. 30,31).

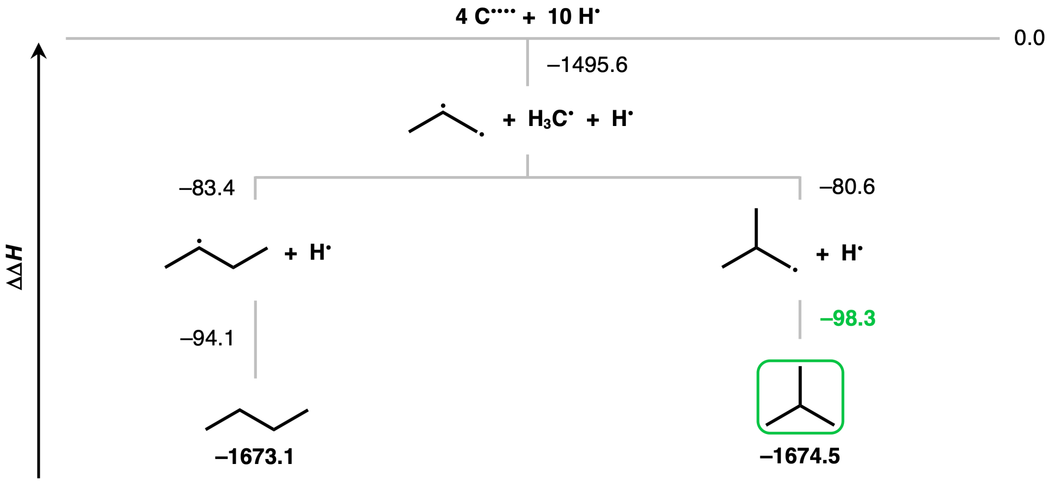

Figure S15. Bond enthalpy (in kcal mol^{-1}) of the step-by-step formation of the C_4H_{10} isomers relative to the atoms in their valence configuration (*i.e.*, C•••• and H•). The relative stability determining step is highlighted in green. Computed at (U)BLYP-D3(BJ)/TZ2P.

Table S5. Experimental and computational enthalpies (in kcal mol^{-1}) of the $C_4H_9^{\bullet}$ radical isomers.

	Experimental [a]				Computational [b]		
**Systems**	**ΔH_f**	**RSE [c]**	**ΔH_{rad}**	**ΔH_{BDE} [d]**	**RSE [c]**	**ΔH_{rad}**	**ΔH_{BDE} [d]**
**$PrH_2C^{\bullet}$**	19.3	3.7	–376.4	–101.4	4.8<br>(3.7)	–389.4<br>(–388.3)	–97.9<br>(–99.9)
**$EtMeHC^{\bullet}$**	16.5	6.5	–366.8	–98.6	8.6<br>(6.5)	–378.0<br>(–379.7)	–94.1<br>(–97.1)
**$Me_3C^{\bullet}$**	11.5	9.4	–361.6	–95.7	11.5<br>(8.4)	–369.9<br>(–373.4)	–91.2<br>(–95.2)

[a] Estimated from the experimental heats of formation ΔH_f in Tables S1 and S4. Heat of formation of gaseous $H^{\bullet}$ is 52.1 kcal mol^{-1}. [b] Computed at (U)BLYP-D3(BJ)/TZ2P (and at (U)M06-2X/TZ2P in parentheses), enthalpies ΔH at 298.15 K and 1 atm. [c] Radical stabilization energy (RSE) calculated using the $R_3C^{\bullet} + H_3C–H \rightarrow R_3C–H + H_3C^{\bullet}$ isodesmic reaction. [d] Bond dissociation enthalpies ΔH_{BDE} associated with the R_3C–H bond.

Supporting Data 1 | Cartesian Coordinates

Table S6. Cartesian coordinates (in Å), ADF total energies (in kcal mol^{-1}), and the number of imaginary frequencies of all radical species in this study, computed at UBLYP-D3(BJ)/TZ2P.

H$^{\bullet}$
E = -22.1
H = -20.7

H	0.00000000	0.00000000	0.00000000

C$^{\bullet\bullet\bullet\bullet}$
E = 81.4
H = 82.8

C	0.00000000	0.00000000	0.00000000

HC$^{\bullet\bullet\bullet}$
E = -115.0
H = -108.7
N_{imag} = 0

C	0.00000000	0.00000000	-0.55161600
H	0.00000000	0.00000000	0.55161600

$H_2C^{\bullet\bullet}$
E = -264.9
H = -252.0
N_{imag} = 0

C	0.00000000	0.00000000	0.27034100
H	0.00000000	-1.00460800	-0.13517100
H	0.00000000	1.00460800	-0.13517100

MeHC$^{\bullet\bullet}$
E = -638.0
H = -606.6
N_{imag} = 0

H	-1.84786900	0.80573200	0.89062800
H	-1.84786900	0.80573200	-0.89062800
H	-0.82564000	1.94666700	0.00000000
C	-0.09562400	-0.04910400	0.00000000
H	-0.01348000	-1.13199600	0.00000000
C	-1.19937600	0.91146200	0.00000000

$Me_2C^{\bullet\bullet}$
E = -1011.0
H = -961.3
N_{imag} = 0

H	-1.72148200	0.54068700	1.53285200
H	-0.05708500	0.37632000	2.12303800
H	-0.92635500	-1.05264600	1.53315300
C	-0.24237000	0.28348700	0.00000000
C	-0.75593000	0.02743600	1.35019200
C	-0.75593000	0.02743600	-1.35019200
H	-1.72148200	0.54068700	-1.53285200
H	-0.92635500	-1.05264600	-1.53315300
H	-0.05708500	0.37632000	-2.12303800

$H_3C^•$
E = -404.4
H = -383.6
N_{imag} = 0

C	0.00000000	0.00000000	0.00000000
H	0.54195500	-0.93869400	0.00000000
H	0.54195500	0.93869400	0.00000000
H	-1.08391100	0.00000000	0.00000000

$MeH_2C^•$
E = -773.3
H = -734.1
N_{imag} = 0

H	0.56052500	1.31262600	-0.92993900
H	-0.86142500	-0.76796900	-0.89096600
H	-0.86142500	-0.76796900	0.89096600
H	0.55155100	-1.33399600	0.00000000
C	0.30086500	0.81457900	0.00000000
C	-0.25061700	-0.56989800	0.00000000
H	0.56052500	1.31262600	0.92993900

$EtH_2C^•$
E = -1139.2
H = -1081.8
N_{imag} = 0

H	0.73590800	1.23929100	-0.93765100
H	-1.04786400	-0.52426600	-0.88057800
C	-1.12990000	-0.78268700	1.28264200
H	0.33712200	-1.35045200	-0.20591300
C	0.40188400	0.77831100	-0.01202600
C	-0.35731700	-0.50499400	-0.02213000
H	0.73374300	1.21968800	0.92486800
H	-0.44718700	-0.80157900	2.14168500
H	-1.64569500	-1.74928700	1.23999500
H	-1.87878800	-0.00280200	1.46632400

$PrH_2C^•$
E = -1505.3
H = -1429.7
N_{imag} = 0

C	1.92042000	0.12596800	-0.01987300
H	-2.14133000	-1.09937900	0.46133000
H	-0.53522700	1.21725900	-0.80631700
H	-2.85349400	0.45350500	-0.27940600
C	-0.62055800	0.49708100	0.02343000
C	-1.98051800	-0.11238400	0.03372700
H	-0.47130900	1.10650200	0.93963500
C	0.53065800	-0.52874700	-0.05702300
H	0.41985600	-1.11699700	-0.97844300
H	0.43231900	-1.23800600	0.77758400
H	2.06368400	0.69577300	0.90770600
H	2.05205100	0.82038000	-0.86011800
H	2.71729500	-0.62506100	-0.07879800

$Me_2HC^•$
E = -1143.2
H = -1085.9
N*imag* = 0

H	-0.03238200	0.56241500	-2.15523500
H	-0.03238200	0.56241500	2.15523500
H	1.05722100	-0.57906500	1.33062300
H	-0.66066700	-0.94161800	1.45583700
C	-0.17770300	0.58602000	0.00000000
C	0.05801700	-0.11346400	1.29740000
C	0.05801700	-0.11346400	-1.29740000
H	-0.66667600	1.55744500	0.00000000
H	-0.66066700	-0.94161800	-1.45583700
H	1.05722100	-0.57906500	-1.33062300

$EtMeHC^•$
E = -1509.1
H = -1433.5
N*imag* = 0

C	0.40183700	1.85817000	0.35380700
H	-1.33179100	-2.01538700	0.22123000
H	0.25231600	-2.82818100	0.22752900
H	-0.41261500	-2.15988600	-1.27348600
C	0.35372700	-0.67485200	0.10354700
C	-0.30768900	-1.98125000	-0.18501900
H	1.40850300	-0.65980300	0.37457800
C	-0.32723800	0.61993200	-0.19864300
H	-1.36164600	0.59130300	0.18757700
H	-0.44775400	0.73391700	-1.29618900
H	1.41860500	1.92539200	-0.05365700
H	0.48141900	1.80886800	1.44673900
H	-0.12767500	2.78177400	0.09198800

$PrMeHC^•$
E = -1875.2
H = -1781.4
N*imag* = 0

H	1.17698800	-1.16402200	-0.95682400
C	1.21208100	-0.56754700	-0.03434300
H	-0.01687000	0.89935300	0.98203200
H	-0.09953700	1.00790900	-0.76212200
C	-1.33666400	-0.52214200	0.06855900
C	-0.07406800	0.27502200	0.06485200
H	-1.28867700	-1.57535800	0.34172900
C	-2.66890200	0.13875400	-0.05704500
H	-2.91623400	0.74424400	0.83739100
H	-3.47974100	-0.58683800	-0.18834200
H	-2.69447900	0.84070200	-0.90647500
H	1.23199900	-1.28563500	0.79823100
C	2.49205100	0.28262500	-0.01375900
H	2.56324900	0.86635600	0.91353000
H	2.50922600	0.99003200	-0.85336800
H	3.38957800	-0.34346100	-0.08548400

$Et_2HC^•$
E = -1875.0
H = -1781.2
N_{imag} = 0

C	0.34565300	-0.19812900	2.52677300
C	0.34565300	-0.19812900	-2.52677300
H	-0.35623000	1.44072400	-1.27606300
H	-1.50285600	0.13045200	-1.44107500
C	0.04236100	-0.22210900	0.00000000
C	-0.42122800	0.33908800	-1.30419300
C	-0.42122800	0.33908800	1.30419300
H	0.57367800	-1.17468200	0.00000000
H	-0.04291300	0.22827100	-3.45911900
H	0.25724300	-1.28999200	-2.59283300
H	1.41231200	0.04798200	-2.45731300
H	-1.50285600	0.13045200	1.44107500
H	-0.35623000	1.44072400	1.27606300
H	-0.04291300	0.22827100	3.45911900
H	1.41231200	0.04798200	2.45731300
H	0.25724300	-1.28999200	2.59283300

$Pr_2HC^•$
E = -2607.2
H = -2477.0
N_{imag} = 0

H	0.24776100	-0.46299700	-4.71002900
C	0.06728500	-0.55964500	2.52992800
H	-1.03671500	0.96268300	1.45600100
H	0.69819800	1.09962300	1.27245800
C	-0.19176400	-0.36651800	0.00000000
C	-0.11823300	0.35625700	1.30425200
H	-0.49934600	-1.41291700	0.00000000
C	-0.11823300	0.35625700	-1.30425200
H	-1.03671500	0.96268300	-1.45600100
C	0.06728500	-0.55964500	-2.52992800
H	0.69819800	1.09962300	-1.27245800
H	0.94706800	0.93037400	-3.86606300
H	-0.81193000	0.77916900	-4.01814600
H	-0.81193000	0.77916900	4.01814600
H	0.94706800	0.93037400	3.86606300
H	0.24776100	-0.46299700	4.71002900
H	-0.75515300	-1.28909500	2.55667500
C	0.11534400	0.21355700	3.85711900
H	0.99193000	-1.14020800	2.40405000
H	0.99193000	-1.14020800	-2.40405000
C	0.11534400	0.21355700	-3.85711900
H	-0.75515300	-1.28909500	-2.55667500

$Me_3C^•$
E = -1513.4
H = -1437.9
N_{imag} = 0

H	1.95054200	0.89112000	0.47358100
H	-1.74700300	-1.24365900	0.47358100
H	-0.20353900	-2.13477900	0.47358100
H	-0.88770500	-1.53755100	-1.04330600
C	0.00000000	0.00000000	0.20123400
C	-0.74379000	-1.28828200	0.02906700
C	1.48758000	0.00000000	0.02906700

C	-0.74379000	1.28828200	0.02906700
H	-1.74700300	1.24365900	0.47358100
H	-0.88770500	1.53755100	-1.04330600
H	-0.20353900	2.13477900	0.47358100
H	1.77541100	0.00000000	-1.04330600
H	1.95054200	-0.89112000	0.47358100

$EtMe_2C^•$
E = -1879.0
H = -1785.1
N_{imag} = 0

C	1.88428700	-0.00682700	0.57099800
H	-1.04275400	-1.51121200	1.17315900
H	-0.76809400	-2.13787800	-0.45883400
H	-2.26516400	-1.25232600	-0.07112300
C	-0.52104200	0.00310800	-0.29574000
C	-1.17923200	-1.28437700	0.09612800
C	0.93411500	0.00128600	-0.66299800
C	-1.17288500	1.28998200	0.10853500
H	-2.25891500	1.26502100	-0.05931800
H	-0.75729500	2.14679500	-0.43794000
H	-1.03562600	1.50557400	1.18777300
H	1.16721500	0.88808300	-1.27128200
H	1.16290000	-0.88089700	-1.27957100
H	2.93499900	-0.00787400	0.25434700
H	1.71384500	-0.89656200	1.18879900
H	1.71814100	0.87785800	1.19716400

$PrMe_2C^•$
E = -2245.3
H = -2133.2
N_{imag} = 0

C	1.32547400	0.00173300	-0.52377500
H	-1.35059500	2.14026500	0.43785200
H	-2.84298600	1.25309000	0.03610900
H	-1.60229300	1.50012400	-1.19260500
C	-1.10323700	-0.00207800	0.29628000
C	-1.75463300	1.28249300	-0.11571000
C	0.34396600	0.00206900	0.68778200
C	-1.74892200	-1.29145700	-0.10962500
H	-1.59613900	-1.51319000	-1.18562300
H	-2.83733200	-1.26636800	0.04261200
H	-1.34068100	-2.14487400	0.44756100
H	0.57022400	-0.88110100	1.30571500
H	0.56620100	0.88888200	1.30194900
C	2.79930500	0.00592000	-0.08948200
H	1.11900600	0.88087800	-1.14946100
H	1.12293000	-0.88091600	-1.14579900
H	3.02902800	0.89364600	0.51436300
H	3.47261600	0.00571300	-0.95552200
H	3.03302000	-0.87835300	0.51788700

$Et_3C^•$
E = -2610.5
H = -2479.9
N_{imag} = 0

C	0.06770700	2.18037400	0.72874000
C	1.85453200	-1.14733900	0.73022900

H	2.09991100	0.37389200	-0.80271000
H	1.58673200	-1.20019500	-1.41351200
C	-0.00004600	-0.00030300	-0.61981100
C	1.42500300	-0.46972600	-0.60061900
C	-0.30617800	1.46852900	-0.60101800
C	-1.11894800	-0.99990900	-0.60054900
H	-1.83152300	-0.77597200	-1.41458500
C	-1.92215400	-1.03165200	0.72932000
H	-0.72527700	-2.00633900	-0.80093600
H	-1.37454900	1.63087800	-0.80166700
H	0.24424800	1.97339300	-1.41509000
H	1.13788300	2.07420700	0.94157300
H	-0.48656800	1.75020500	1.57074200
H	-0.16087000	3.25272300	0.67446600
H	2.89746600	-1.48577400	0.67650800
H	1.22736800	-2.02059900	0.94448200
H	1.75921400	-0.45082800	1.57108000
H	-2.73665900	-1.76567400	0.67536300
H	-2.36507900	-0.05164200	0.94201000
H	-1.27235600	-1.29648000	1.57126600

$Pr_3C^{\bullet}$
E = -3709.7
H = -3524.4
N_{imag} = 0

C	1.18617900	-2.22899100	-0.48115500
C	-2.41684500	0.00475000	-0.05173200
H	-1.22274600	-0.66169300	-1.73446700
H	-1.29881100	1.08383300	-1.55127600
C	0.15538900	0.11663200	-0.29924400
C	-1.18467100	0.13959700	-0.98235500
C	1.25239500	-0.72410300	-0.88190800
C	0.41066400	1.02850100	0.86549100
H	-0.31406900	0.83776000	1.67573200
C	0.33053100	2.54445300	0.51770000
H	1.40355400	0.81854400	1.28587000
H	2.23383900	-0.33152800	-0.57768200
H	1.21698000	-0.67179400	-1.98337700
C	1.43182700	-2.46605400	1.01670800
H	1.93216400	-2.78163700	-1.07058200
H	0.20286100	-2.62885200	-0.76379100
H	-2.42130900	0.83069000	0.67226000
C	-2.46763800	-1.33719500	0.69544800
H	-3.32534900	0.12411700	-0.65806300
H	-0.68057800	2.77756800	0.15702600
C	1.36550600	2.97515300	-0.53316400
H	0.47222500	3.12324700	1.44125200
H	2.42625900	-2.10598200	1.31231600
H	1.37254300	-3.53234600	1.26627200
H	0.69182800	-1.93601800	1.62746700
H	-1.58771400	-1.46694700	1.33587800
H	-2.48798300	-2.17777000	-0.01055300
H	-3.36063900	-1.40770600	1.32810600
H	1.28909700	4.04684000	-0.75311000
H	1.22500600	2.42613600	-1.47235900
H	2.38680600	2.77538200	-0.18281600

$H_3CC^{\bullet\bullet}H_2C^{\bullet}$
E = -872.0
H = -830.6
N_{imag} = 0

C	2.01171800	0.05442500	-0.04314100
H	2.32873800	0.58603300	0.87595300
H	-0.82679300	1.27165200	-0.82305900
H	2.28156400	0.69183900	-0.90842600
C	-0.56225400	0.68233000	0.06222100
H	2.62313400	-0.85862600	-0.11343100
H	-0.77796900	1.16208600	1.02362600
C	0.57887600	-0.28058500	-0.02510100

$H_3CHC^{\bullet}H_2C^{\bullet}$
E = -1015.3
H = -966.5
N_{imag} = 0

H	-1.24816000	-2.23981700	0.31773200
H	0.42060100	-2.84859500	0.18507800
H	-0.46337200	-2.28713300	-1.25605400
C	0.24767100	-0.69862200	0.03389700
C	-0.27782400	-2.09033100	-0.18622300
H	1.22043700	-0.58625800	0.52025000
C	-0.48999900	0.48152700	-0.37371600
H	-1.20821800	0.97317800	0.28811200
H	-0.37532200	0.92184300	-1.36795300

$H_2C^{\bullet}H_2CH_2C^{\bullet}$
E = -1011.3
H = -962.9
N_{imag} = 0

H	-2.09295500	-0.73243500	1.17137200
H	-0.87031800	0.75446000	-1.22585300
H	-2.81196000	0.80259800	0.39180000
C	-0.68875100	0.35740300	-0.20871600
C	-1.98461300	0.10551500	0.48848500
H	-0.15777700	1.21101500	0.28773700
C	0.22009600	-0.82550000	-0.26849400
H	1.07302100	-0.83717000	-0.94089700
H	0.09365500	-1.65705300	0.41908400

$H_3CH_2CHC^{\bullet}H_2C^{\bullet}$
E = -1381.3
H = -1314.9
N_{imag} = 0

C	1.90535800	0.17927000	0.04998300
H	-2.46270900	-0.85816500	0.94163400
C	-0.62625900	0.25843100	0.34396300
C	-1.99227300	-0.20469800	0.20189000
H	-0.42185800	1.19691300	0.86910400
C	0.55236400	-0.51119800	-0.19278300
H	0.41108000	-0.69003500	-1.27418400
H	0.56347500	-1.52269600	0.25364200
H	2.08434900	0.32156700	1.12300200
H	1.93217400	1.16570700	-0.42936300
H	2.73085500	-0.41737700	-0.35517200
H	-2.60747800	0.06091600	-0.66196200

$H_3CHC^{\bullet}H_2CH_2C^{\bullet}$
E = -1381.4
H = -1314.9
N_{imag} = 0

C	1.92953100	0.18457600	0.11942600
H	-1.99873300	-1.29234500	-0.01210000
C	-0.64484100	0.50207100	-0.04264800
C	-1.95426100	-0.20764200	0.03276700
H	-0.68249500	1.41653300	0.59318300
C	0.54439900	-0.33361900	0.31149100
H	0.38455700	-1.25627700	0.86573300
H	2.15970200	1.01643800	0.81383100
H	2.07071300	0.59602400	-0.89363300
H	2.68609200	-0.59088600	0.28297900
H	-2.88823900	0.34713100	0.03255400
H	-0.49243300	0.93088700	-1.06170500

$H_2C^{\bullet}H_2CH_2CH_2C^{\bullet}$
E = -1377.5
H = -1310.8
N_{imag} = 0

C	1.88583600	-0.08853800	0.26206100
H	-2.23019200	-1.00564100	0.30154900
C	-0.58436400	0.50408400	0.04133300
C	-1.97479900	-0.01948800	-0.07946700
H	-0.46942900	1.05294500	0.99923400
C	0.50905800	-0.59136700	-0.00934300
H	0.23960500	-1.37285000	0.73139800
H	2.04595200	0.75212000	0.93330100
H	2.75926400	-0.62227400	-0.10138600
H	-2.78322200	0.61356700	-0.43377000
H	-0.38868500	1.25505000	-0.73837200
H	0.47277000	-1.10209400	-0.98298700

$H_3CHC^{\bullet}HC^{\bullet}H_3C$
E = -1385.0
H = -1318.0
N_{imag} = 0

C	0.17094900	1.70661900	0.44477200
H	-0.93062000	-1.53824800	0.82595100
H	0.30336300	-2.72090400	0.31512100
H	-0.90806700	-2.11642000	-0.83583200
C	0.66469800	-0.70048000	-0.37157300
C	-0.25322500	-1.82791400	0.00733700
C	0.17663100	0.64709600	-0.62041700
H	-0.12760900	0.93640700	-1.63149000
H	1.19815100	2.02197200	0.70626700
H	-0.27462600	1.33466200	1.38072900
H	-0.37942300	2.60028400	0.12790700
H	1.72142300	-0.93438100	-0.53634700

$H_3CC^{\bullet\bullet}H_2CH_3C$
E = -1376.7
H = -1308.6
N_{imag} = 0

C	0.50839300	1.83000300	0.34833500
H	-1.34219100	-2.21832000	0.26971800
H	0.33740600	-2.77499000	0.15230100

H	-0.49677200	-2.18590700	-1.29742800
C	0.11508700	-0.65583500	0.07635700
C	-0.36476900	-2.01149100	-0.21065400
C	-0.40410500	0.69539900	-0.17461500
H	-1.40760700	0.80451900	0.28766000
H	-0.56403000	0.84108100	-1.26356000
H	1.49525500	1.78117200	-0.12542700
H	0.64804600	1.74480600	1.43183000
H	0.06678200	2.80936300	0.13090800

$(H_3C)_2HCH_2C^{\bullet}$
E = -1506.2
H = -1430.8
$\boldsymbol{N_{imag}}$ = 0

H	-1.78011800	0.14070000	1.29445500
H	-0.25253500	0.33371000	2.17938200
H	-0.59363600	-1.18243300	1.32109200
C	-0.04339100	0.47062900	0.00000000
C	-0.70835700	-0.09178800	1.27717300
C	-0.70835700	-0.09178800	-1.27717300
H	-1.78011800	0.14070000	-1.29445500
H	-0.59363600	-1.18243300	-1.32109200
H	-0.25253500	0.33371000	-2.17938200
H	-0.23029800	1.56559300	0.00000000
H	1.99489700	0.23562200	-0.93159900
C	1.43469300	0.25533500	0.00000000
H	1.99489700	0.23562200	0.93159900

$(H_3C)_2C^{\bullet}H_2C^{\bullet}$
E = -1384.9
H = -1318.0
$\boldsymbol{N_{imag}}$ = 0

H	2.12351500	0.13106100	-0.68252400
H	-1.91256900	-0.68575200	-0.19355700
H	-0.51734000	-1.72105600	0.21510100
H	-0.75892600	-1.17048300	-1.44756100
C	-0.02369700	0.36617100	-0.10083100
C	-0.84556200	-0.86421600	-0.38575400
C	1.40756900	0.23331100	0.14050500
C	-0.59056700	1.70890500	-0.48322600
H	-1.67002300	1.75860700	-0.28426700
H	-0.45533200	1.91092600	-1.56471400
H	-0.09498700	2.52749600	0.05270400
H	1.82863700	0.22951300	1.14766300

Table S7. Cartesian coordinates (in Å), ADF total energies (in kcal mol⁻¹), and the number of imaginary frequencies of all alkanes (*i.e.*, the parent molecules) in this study, computed at BLYP-D3(BJ)/TZ2P.

CH_4
E = -536.7
H = -507.0
N_{imag} = 0

C	0.00000000	0.00000000	0.00000000
H	0.63181200	-0.63181200	0.63181200
H	-0.63181200	0.63181200	0.63181200
H	0.63181200	0.63181200	-0.63181200
H	-0.63181200	-0.63181200	-0.63181200

C_2H_6
E = -900.79
H = -852.50
N_{imag} = 0

H	0.88539000	0.51118000	-1.16709400
H	0.88539000	-0.51118000	1.16709400
H	-0.88539000	-0.51118000	1.16709400
H	0.00000000	1.02236000	1.16709400
C	0.00000000	0.00000000	-0.76906200
C	0.00000000	0.00000000	0.76906200
H	-0.88539000	0.51118000	-1.16709400
H	0.00000000	-1.02236000	-1.16709400

***n*-C_3H_8**
E = -1266.9
H = -1200.4
N_{imag} = 0

H	0.00000000	2.18136400	-0.39601900
H	-0.87932900	0.00000000	-1.28059600
C	0.00000000	-1.28104500	0.23059600
H	0.87932900	0.00000000	-1.28059600
C	0.00000000	1.28104500	0.23059600
C	0.00000000	0.00000000	-0.62075300
H	0.88590600	1.32238200	0.87819800
H	0.88590600	-1.32238200	0.87819800
H	0.00000000	-2.18136400	-0.39601900
H	-0.88590600	-1.32238200	0.87819800
H	-0.88590600	1.32238200	0.87819800

***n*-C_4H_{10}**
E = -1633.0
H = -1548.3
N_{imag} = 0

C	1.96681300	-0.14015900	0.00000900
H	-2.10487200	0.77391000	0.88596300
H	-0.47377200	-1.16503000	0.87972500
H	-2.10487400	0.77394900	-0.88591900
C	-0.57442300	-0.51178600	-0.00000900
C	-1.96681300	0.14015900	0.00000900
H	-0.47378500	-1.16500300	-0.87976400
C	0.57442300	0.51178600	-0.00000900
H	0.47377200	1.16503000	0.87972500
H	0.47378500	1.16500300	-0.87976400
H	2.10487400	-0.77394900	-0.88591900

H	2.10487200	-0.77391000	0.88596300
H	2.76359200	0.61360000	-0.00000600
H	-2.76359200	-0.61360000	-0.00000600

***i*-C$_4$H$_{10}$**
E = -1634.1
H = -1549.8
N$_{imag}$ = 0

H	2.00112800	0.88767300	0.29510200
H	-1.76931200	-1.28919100	0.29510200
H	-0.23181600	-2.17686500	0.29510200
H	-0.75924700	-1.31505400	-1.16318000
C	0.00000000	0.00000000	0.40689800
C	-0.73316600	-1.26988100	-0.06539800
C	1.46633300	0.00000000	-0.06539800
C	-0.73316600	1.26988100	-0.06539800
H	-1.76931200	1.28919100	0.29510200
H	-0.75924700	1.31505400	-1.16318000
H	-0.23181600	2.17686500	0.29510200
H	1.51849400	0.00000000	-1.16318000
H	2.00112800	-0.88767300	0.29510200
H	0.00000000	0.00000000	1.50822300

***n*-C$_5$H$_{12}$**
E = -1999.0
H = -1896.1
N$_{imag}$ = 0

C	0.31626100	-0.20923600	2.56711800
C	0.31626100	-0.20923600	-2.56711800
H	-0.43984200	1.36938900	-1.28625900
H	-1.42980300	-0.08513600	-1.28638000
C	0.30679600	-0.20301500	0.00000000
C	-0.38862900	0.27038200	-1.28749100
C	-0.38862900	0.27038200	1.28749100
H	0.35884800	-1.30312100	0.00000000
H	-0.20155100	0.14324400	-3.46750000
H	0.35296500	-1.30580800	-2.60902100
H	1.34983400	0.15892900	-2.60889700
H	-1.42980300	-0.08513600	1.28638000
H	-0.43984200	1.36938900	1.28625900
H	-0.20155100	0.14324400	3.46750000
H	1.34983400	0.15892900	2.60889700
H	0.35296500	-1.30580800	2.60902100
H	1.34936100	0.15195000	0.00000000

***i*-C$_5$H$_{12}$**
E = -1999.6
H = -1897.0
N$_{imag}$ = 0

C	1.81522400	0.85514500	0.58408000
H	-2.27780300	-1.54204800	0.08807100
H	-0.75471000	-2.43558800	0.27248600
H	-1.13522900	-1.63594600	-1.26479400
C	-0.51671600	-0.25681600	0.31309900
C	-1.21205600	-1.54296100	-0.17250200
C	0.99406100	-0.30095200	-0.01160800
C	-1.20593000	0.98748500	-0.28000300
H	-2.27742200	0.99076500	-0.04465500

H	-1.10354900	0.99880000	-1.37434300
H	-0.77780000	1.91846400	0.10754300
H	1.11881000	-0.31693500	-1.10569100
H	1.40035600	-1.25385800	0.35715600
H	2.88422800	0.72459400	0.37670800
H	1.68994300	0.90396600	1.67406300
H	1.51549200	1.82472100	0.16990500
H	-0.61817200	-0.21026900	1.40980700

***t*-C_5H_{12}**
E = -2001.7
H = -1899.6
N_{imag} = 0

H	1.99832900	0.88722500	0.27674100
H	-1.76784000	-1.28653100	0.27693200
H	-0.23074900	-2.17418800	0.27686800
H	-0.74315600	-1.28670800	-1.17240300
C	0.00001800	0.00000000	0.43979300
C	-0.72867200	-1.26171300	-0.07526400
C	1.45700700	-0.00020500	-0.07535500
C	-0.72831900	1.26191800	-0.07526300
H	-1.76750000	1.28699100	0.27687500
H	-0.74273400	1.28695300	-1.17240200
H	-0.23018200	2.17425400	0.27692700
H	1.48579300	-0.00024100	-1.17249700
H	1.99809800	-0.88775500	0.27679400
C	-0.00000600	-0.00000000	1.98515700
H	0.51217300	-0.88760300	2.37804700
H	-1.02480800	0.00021600	2.37797400
H	0.51254700	0.88738700	2.37804700

***i*-C_6H_{14}**
E = -2365.7
H = -2244.9
N_{imag} = 0

C	1.77777400	0.86886000	0.70155000
H	-2.27622100	-1.55789100	0.01035400
H	-0.75733900	-2.43695900	0.28032400
H	-1.06378500	-1.65129800	-1.28044700
C	-0.53991400	-0.25587100	0.31748500
C	-1.19832500	-1.55148700	-0.19438400
C	0.98503600	-0.28919200	0.07107100
C	-1.20920800	0.97783200	-0.31942000
H	-2.29218700	0.96964200	-0.14419300
H	-1.04602800	0.98541700	-1.40638100
H	-0.81367100	1.91535300	0.08643300
H	1.16876900	-0.30643700	-1.01556300
H	1.38158500	-1.23835700	0.46271000
C	3.29600000	0.72832800	0.49990100
H	1.55289300	0.91387500	1.77782300
H	1.44532600	1.82517000	0.27650300
H	3.84088000	1.56310300	0.95738900
H	3.55045300	0.70729100	-0.56799200
H	3.66905500	-0.20210500	0.94779700
H	-0.69909900	-0.20353600	1.40688500

***n*-C_7H_{16}**
E = -2731.1
H = -2591.9
N_{imag} = 0

C	1.28601800	-0.31446300	0.08682800
C	-2.57303600	0.49758000	-0.15505300
C	-3.85217100	-0.34926500	-0.05034300
C	2.57277400	0.52195900	-0.01525300
C	-0.00012400	0.51013800	-0.08523800
C	-1.28583500	-0.32664600	0.01703800
C	3.85232400	-0.31287100	0.15826500
H	1.31135500	-1.11073400	-0.67332800
H	1.26279400	-0.82421900	1.06259900
H	-2.59590500	1.29299200	0.60469800
H	-2.54781300	1.00590500	-1.13044000
H	-4.75223400	0.26469000	-0.17691000
H	-3.91821100	-0.84344000	0.92793400
H	-3.86980400	-1.13274400	-0.81940300
H	2.59552200	1.03067200	-0.99048400
H	2.54701000	1.31703000	0.74473600
H	-0.02453900	1.30622000	0.67497000
H	0.02395400	1.01962800	-1.06099000
H	-1.31079900	-0.83629100	0.99283800
H	-1.26229300	-1.12302800	-0.74307300
H	3.91893600	-1.09547000	-0.60899200
H	3.86998800	-0.80725700	1.13849800
H	4.75208900	0.30961400	0.08077100

C_7H_{16}
E = -2730.3
H = -2591.1
N_{imag} = 0

C	2.44912900	0.93875400	0.60280500
C	-2.04379500	-1.65929800	0.57319500
H	-0.00513200	-2.36239000	0.43267000
H	-0.57409300	-1.55676600	-1.01761500
C	0.11773600	-0.21282500	0.54746000
C	-0.59528900	-1.50435700	0.08037700
C	1.59726600	-0.23645400	0.09465600
C	-0.62726500	1.08023600	0.12572900
H	-1.63201200	1.06756500	0.56719800
C	-0.74748700	1.30485200	-1.39240500
H	-0.11929100	1.94222800	0.57682300
H	1.64285100	-0.27449500	-1.00318200
H	2.04311200	-1.17801300	0.44580900
H	3.50316700	0.80361700	0.33148300
H	2.39332100	1.02166100	1.69648000
H	2.12075800	1.89518300	0.17969300
H	-2.09879700	-1.57556600	1.66685000
H	-2.44962000	-2.63874800	0.29239800
H	-2.70474300	-0.89532500	0.14761600
H	0.23616200	1.37672000	-1.87154600
H	-1.28519500	2.23722600	-1.60237300
H	-1.29592900	0.49092100	-1.88131400
H	0.11763700	-0.22507500	1.65065500

$C_{10}H_{22}$
E = -3828.8
H = -3634.9
N_{imag} = 0

C	2.51936100	0.90505000	0.55235400
C	-1.95553400	-1.74060400	0.82068600
H	0.06829500	-2.41440400	0.49843600
H	-0.61855800	-1.56050200	-0.87180900
C	0.19290900	-0.26781600	0.68139600
C	-0.55047000	-1.54599300	0.22624900
C	1.62487200	-0.25980100	0.09581300
C	-0.59465400	1.03405400	0.38546300
H	-1.55650200	0.99486800	0.91466200
C	-0.86048800	1.33257600	-1.10078100
H	-0.05378300	1.88347100	0.82442400
H	1.57280300	-0.26480500	-1.00321300
H	2.11204400	-1.20600200	0.37586600
C	3.96456400	0.77637300	0.04236400
H	2.51968700	0.95167800	1.65177800
H	2.09965300	1.85828500	0.20442100
H	-1.89899700	-1.66073000	1.91672700
C	-2.57933000	-3.09236000	0.43487300
H	-2.61866800	-0.93127600	0.48734800
H	0.09192600	1.39564800	-1.64449600
C	-1.64637300	2.63900700	-1.30279600
H	-1.41778000	0.50153900	-1.55406800
H	4.58435900	1.61850300	0.37367600
H	3.99299000	0.75095300	-1.05504700
H	4.43090000	-0.14821900	0.40713000
H	-1.96164000	-3.92770200	0.78995900
H	-2.66877300	-3.18677600	-0.65546000
H	-3.58183900	-3.20971400	0.86418300
H	-1.09827000	3.49585800	-0.88926700
H	-2.62027100	2.59444800	-0.79767300
H	-1.82877100	2.83897900	-2.36573000
H	0.29340500	-0.32653900	1.77818900

Table S8. Cartesian coordinates (in Å), ADF total energies (in kcal mol^{-1}), and the number of imaginary frequencies of all radical species in this study, computed at UM06-2X/TZ2P.

H$^{\bullet}$
E = -51.7
H = -50.2

H	0.00000000	0.00000000	0.00000000

C$^{\bullet\bullet\bullet\bullet}$
E = -1.3
H = 0.2

C	0.00000000	0.00000000	0.00000000

HC$^{\bullet\bullet\bullet}$
E = -227.8
H = -221.3
N_{imag} = 0

C	0.00000000	0.00000000	-0.54545200
H	0.00000000	0.00000000	0.54545200

H$_3$C$^{\bullet}$
E = -571.1
H = -549.9
N_{imag} = 0

C	0.00000000	0.00000000	0.00000000
H	0.53811100	-0.93203500	0.00000000
H	0.53811100	0.93203500	0.00000000
H	-1.07622200	0.00000000	0.00000000

MeH$_2$C$^{\bullet}$
E = -1080.3
H = -1039.7
N_{imag} = 0

H	0.62214000	1.27251200	-0.92319500
H	-0.89226500	-0.72408300	-0.88345200
H	-0.89226500	-0.72408300	0.88345200
H	0.49240600	-1.34048800	0.00000000
C	0.32396700	0.80102200	0.00000000
C	-0.27612200	-0.55739200	0.00000000
H	0.62214000	1.27251200	0.92319500

EtH$_2$C$^{\bullet}$
E = -1587.3
H = -1528.0
N_{imag} = 0

H	0.76802100	1.20864800	-0.91211000
H	-1.14634300	-0.40968800	-0.84436900
C	-1.11320300	-0.76650600	1.28642800
H	0.21073800	-1.35529900	-0.30341200
C	0.42568100	0.73987700	-0.00242700
C	-0.41041600	-0.48838600	-0.03981400
H	0.83338100	1.09311700	0.93365300
H	-0.38427500	-0.88043800	2.08926100
H	-1.70697900	-1.67807600	1.23863700
H	-1.77469900	0.05797400	1.55136900

$PrH_2C^•$
E = -2094.6
H = -2016.5
N*imag* = 0

C	1.90101200	0.13314900	-0.01876500
H	-2.10122200	-1.11464200	0.42957500
H	-0.51972300	1.19702600	-0.81764900
H	-2.83559800	0.44299000	-0.24643600
C	-0.60884800	0.49571000	0.01767100
C	-1.95779200	-0.12656300	0.01674800
H	-0.47505700	1.10832100	0.92178600
C	0.52705300	-0.52372300	-0.04607300
H	0.41662600	-1.12180900	-0.95279500
H	0.42897800	-1.21482100	0.79434300
H	2.03528200	0.71498600	0.89410500
H	2.02374000	0.81149800	-0.86410600
H	2.69939600	-0.60622700	-0.06497200

$Me_2HC^•$
E = -1590.5
H = -1531.1
N*imag* = 0

H	0.01250400	0.56121100	-2.14053800
H	0.01250400	0.56121100	2.14053800
H	1.04221900	-0.60297900	1.29766900
H	-0.67762700	-0.90472100	1.44943500
C	-0.16303700	0.59632300	0.00000000
C	0.06509900	-0.11220300	1.28697600
C	0.06509900	-0.11220300	-1.28697600
H	-0.72135600	1.52106200	0.00000000
H	-0.67762700	-0.90472100	-1.44943500
H	1.04221900	-0.60297900	-1.29766900

$EtMeHC^•$
E = -2097.5
H = -2019.3
N*imag* = 0

C	0.39629300	1.84092400	0.34513400
H	-1.32334900	-1.98875000	0.21382300
H	0.24423600	-2.80536700	0.24665400
H	-0.38482500	-2.14234100	-1.25867700
C	0.35397200	-0.66486200	0.11598000
C	-0.30289000	-1.96555200	-0.17796300
H	1.41723400	-0.64099300	0.31629500
C	-0.33878500	0.61619800	-0.18947700
H	-1.35586400	0.58296100	0.21663000
H	-0.47100900	0.71794900	-1.27659300
H	1.39703500	1.90488600	-0.08291800
H	0.50049700	1.78355800	1.42852500
H	-0.13254600	2.76138700	0.10258800

$PrMeHC^•$
E = -2604.7
H = -2507.7
N*imag* = 0

H	1.16835200	-1.18270100	-0.91859500
C	1.20210700	-0.56476100	-0.01892600
H	-0.02352200	0.91215400	0.95026600

H	-0.09238500	0.97839900	-0.78954200
C	-1.32052600	-0.52895000	0.06166400
C	-0.06947600	0.27557400	0.05253200
H	-1.27170200	-1.55330700	0.40740000
C	-2.64546600	0.13329400	-0.06456700
H	-2.89084200	0.72155600	0.82967600
H	-3.44993400	-0.58563600	-0.20946200
H	-2.65722900	0.83377700	-0.90390300
H	1.21967900	-1.25356100	0.82881300
C	2.46567200	0.28601000	-0.01921600
H	2.52609200	0.89201700	0.88580300
H	2.47605400	0.96518500	-0.87273300
H	3.36312600	-0.32905700	-0.07064600

$Et_2HC^{\bullet}$
E = -2604.5
H = -2507.4
N_{imag} = 0

C	0.33471900	-0.20521200	2.50194300
C	0.33471900	-0.20521200	-2.50194300
H	-0.33670600	1.44242600	-1.27599300
H	-1.48946600	0.14462000	-1.41019400
C	0.06718900	-0.20101000	0.00000000
C	-0.41521500	0.34989600	-1.29514200
C	-0.41521500	0.34989600	1.29514200
H	0.57586300	-1.15850800	0.00000000
H	-0.04361800	0.21265900	-3.43362800
H	0.23097000	-1.28939500	-2.55257700
H	1.39779200	0.02476500	-2.43250700
H	-1.48946600	0.14462000	1.41019400
H	-0.33670600	1.44242600	1.27599300
H	-0.04361800	0.21265900	3.43362800
H	1.39779200	0.02476500	2.43250700
H	0.23097000	-1.28939500	2.55257700

$Pr_2HC^{\bullet}$
E = -3618.9
H = -3484.3
N_{imag} = 0

H	0.22029900	-0.45244000	-4.67336300
C	0.05991000	-0.55816800	2.50742400
H	-1.01393400	0.95944500	1.42824000
H	0.71735200	1.08371200	1.27352300
C	-0.15646000	-0.37261000	0.00000000
C	-0.10253100	0.35544600	1.29577400
H	-0.48504400	-1.40584900	0.00000000
C	-0.10253100	0.35544600	-1.29577400
H	-1.01393400	0.95944500	-1.42824000
C	0.05991000	-0.55816800	-2.50742400
H	0.71735200	1.08371200	-1.27352300
H	0.93963700	0.91637500	-3.82820300
H	-0.81118800	0.78828400	-3.96412600
H	-0.81118800	0.78828400	3.96412600
H	0.93963700	0.91637500	3.82820300
H	0.22029900	-0.45244000	4.67336300
H	-0.76666300	-1.27219600	2.52607000
C	0.10500200	0.21409500	3.81963500
H	0.97286800	-1.14532300	2.38852400
H	0.97286800	-1.14532300	-2.38852400

C	0.10500200	0.21409500	-3.81963500
H	-0.76666300	-1.27219600	-2.52607000

Me$_3$C$^•$
E = -2101.1
H = -2022.9
***N**$_{imag}$* = 0

H	1.94178300	0.88509700	0.46634100
H	-1.73740800	-1.23908500	0.46634100
H	-0.20437500	-2.12418200	0.46634100
H	-0.86798900	-1.50340100	-1.03930300
C	0.00000000	0.00000000	0.23048800
C	-0.73855600	-1.27921700	0.02979300
C	1.47711200	0.00000000	0.02979300
C	-0.73855600	1.27921700	0.02979300
H	-1.73740800	1.23908500	0.46634100
H	-0.86798900	1.50340100	-1.03930300
H	-0.20437500	2.12418200	0.46634100
H	1.73597800	0.00000000	-1.03930300
H	1.94178300	-0.88509700	0.46634100

EtMe$_2$C$^•$
E = -2607.8
H = -2510.7
***N**$_{imag}$* = 0

C	1.83252600	-0.00702200	0.56514700
H	-0.98703000	-1.46649900	1.16974500
H	-0.75285300	-2.12940700	-0.44483300
H	-2.23718800	-1.25279200	-0.04281700
C	-0.52235600	0.00342500	-0.33693200
C	-1.15525600	-1.27456600	0.10024200
C	0.93113500	0.00158300	-0.68182200
C	-1.14920300	1.28018700	0.11237800
H	-2.23121000	1.26494300	-0.03098000
H	-0.74265300	2.13826400	-0.42447100
H	-0.98021700	1.46109300	1.18368200
H	1.16984500	0.88269500	-1.28238000
H	1.16548900	-0.87455600	-1.29131500
H	2.88676200	-0.00816100	0.28940200
H	1.64121300	-0.89161400	1.17298800
H	1.64548800	0.87218500	1.18206400

PrMe$_2$C$^•$
E = -3115.1
H = -2999.2
***N**$_{imag}$* = 0

C	1.29158800	0.00129900	-0.50253200
H	-1.33031400	2.13185500	0.41447900
H	-2.80908700	1.25324000	-0.00340900
H	-1.54023300	1.45646100	-1.19822500
C	-1.09993400	-0.00189100	0.32695000
C	-1.72494600	1.27302000	-0.12995500
C	0.34276800	0.00224500	0.70970400
C	-1.71905600	-1.28163500	-0.12442900
H	-1.53372700	-1.46869500	-1.19196500
H	-2.80325200	-1.26641100	0.00229600
H	-1.32027500	-2.13631000	0.42351400
H	0.56963600	-0.87510300	1.32329900

H	0.56575000	0.88362600	1.31892100
C	2.75714400	0.00572000	-0.08655700
H	1.07829900	0.87524200	-1.12203500
H	1.08230900	-0.87679500	-1.11751600
H	2.98686800	0.88817100	0.51205100
H	3.42052600	0.00498100	-0.95072300
H	2.99088400	-0.87254400	0.51663800

Et$_3$C$^•$
E = -3621.5
H = -3486.5
N$_{imag}$ = 0

C	0.07460100	2.12354200	0.72186800
C	1.80110300	-1.12421600	0.72489300
H	2.08598100	0.40611600	-0.77207800
H	1.61750100	-1.15674800	-1.41404000
C	-0.00026000	-0.00033400	-0.65058300
C	1.42446100	-0.44652100	-0.60167800
C	-0.32621000	1.45666100	-0.60306500
C	-1.09904200	-1.01108800	-0.60207900
H	-1.81012500	-0.82336900	-1.41494100
C	-1.87508500	-0.99809500	0.72397700
H	-0.69128100	-2.01033300	-0.77199500
H	-1.39566200	1.60293800	-0.77188600
H	0.19090800	1.97789000	-1.41704700
H	1.14543700	2.01478700	0.89785900
H	-0.44884000	1.66188600	1.55959800
H	-0.15756700	3.18864800	0.71470600
H	2.83949000	-1.45606800	0.71869000
H	1.17117400	-1.99659900	0.90269700
H	1.66309800	-0.43813600	1.56105000
H	-2.68162900	-1.73148100	0.71744400
H	-2.31573100	-0.01631600	0.90127300
H	-1.21246400	-1.22139300	1.56061900

Pr$_3$C$^•$
E = -5144.4
H = -4952.9
N$_{imag}$ = 0

C	1.24469500	-2.19098700	-0.53367200
C	-2.40460100	0.06343300	-0.07334900
H	-1.22109700	-0.71786700	-1.68969800
H	-1.22718800	1.02807700	-1.59462100
C	0.14078900	0.06925300	-0.26204600
C	-1.17170100	0.11519700	-0.98182200
C	1.26856100	-0.69181600	-0.88223000
C	0.36093700	0.97218700	0.90934500
H	-0.37607400	0.76951000	1.69534900
C	0.27707600	2.46559100	0.54501800
H	1.34215100	0.76959900	1.34621500
H	2.22789800	-0.27544000	-0.56102000
H	1.22826400	-0.58821200	-1.97226500
C	1.49236300	-2.44350400	0.94866700
H	1.99832400	-2.71099700	-1.12854100
H	0.27470700	-2.60454200	-0.82160800
H	-2.40089000	0.92261300	0.60131000
C	-2.47610100	-1.22714300	0.73465700
H	-3.30015600	0.16202000	-0.68886000
H	-0.72601600	2.68747000	0.17259000

C	1.31149700	2.86539800	-0.50058400
H	0.41148300	3.06145800	1.44954000
H	2.47039800	-2.06153900	1.24671900
H	1.46143900	-3.50657000	1.18443100
H	0.74079100	-1.94161300	1.55944500
H	-1.61273300	-1.32480500	1.39397000
H	-2.48216600	-2.09491000	0.07257500
H	-3.37551100	-1.26614700	1.34799000
H	1.25147000	3.92691200	-0.73750400
H	1.16689700	2.30512900	-1.42581400
H	2.32179300	2.65683200	-0.14429300

Table S9. Cartesian coordinates (in Å), ADF total energies (in kcal mol^{-1}), and the number of imaginary frequencies of all alkanes (*i.e.*, the parent molecules) in this study, computed at M06-2X/TZ2P.

CH_4
E = -734.3
H = -703.7
N_{imag} = 0

C	0.00000000	0.00000000	0.00000000
H	0.62746200	-0.62746200	0.62746200
H	-0.62746200	0.62746200	0.62746200
H	0.62746200	0.62746200	-0.62746200
H	-0.62746200	-0.62746200	-0.62746200

C_2H_6
E = -1239.6
H = -1189.8
N_{imag} = 0

H	0.87944900	0.50775000	-1.15646300
H	0.87944900	-0.50775000	1.15646300
H	-0.87944900	-0.50775000	1.15646300
H	0.00000000	1.01550000	1.15646300
C	0.00000000	0.00000000	-0.76225600
C	0.00000000	0.00000000	0.76225600
H	-0.87944900	0.50775000	-1.15646300
H	0.00000000	-1.01550000	-1.15646300

***n*-C_3H_8**
E = -1746.9
H = -1678.2
N_{imag} = 0

H	0.00000000	2.16434600	-0.38502000
H	-0.87378500	0.00000000	-1.27691000
C	0.00000000	-1.26429700	0.22868000
H	0.87378500	0.00000000	-1.27691000
C	0.00000000	1.26429700	0.22868000
C	0.00000000	0.00000000	-0.62291400
H	0.88018800	1.29680300	0.87235400
H	0.88018800	-1.29680300	0.87235400
H	0.00000000	-2.16434600	-0.38502000
H	-0.88018800	-1.29680300	0.87235400
H	-0.88018800	1.29680300	0.87235400

***n*-C_4H_{10}**
E = -2254.1
H = -2166.6
N_{imag} = 0

C	1.94410500	-0.13918700	0.00000000
H	-2.07512100	0.76996100	0.88026800
H	-0.46272000	-1.15835800	0.87440700
H	-2.07512100	0.76996100	-0.88026800
C	-0.56586200	-0.51073100	0.00000000
C	-1.94410500	0.13918700	0.00000000
H	-0.46272000	-1.15835800	-0.87440700
C	0.56586200	0.51073100	0.00000000
H	0.46272000	1.15835800	0.87440700
H	0.46272000	1.15835800	-0.87440700
H	2.07512100	-0.76996100	-0.88026800

H	2.07512100	-0.76996100	0.88026800
H	2.74006000	0.60451800	0.00000000
H	-2.74006000	-0.60451800	0.00000000

***i*-C_4H_{10}**
E = -2255.4
H = -2168.3
N_{imag} = 0

H	1.98312600	0.88292600	0.28919900
H	-1.75619900	-1.27597400	0.28919900
H	-0.22692600	-2.15890000	0.28919900
H	-0.74364700	-1.28803400	-1.15521700
C	0.00000000	0.00000000	0.41369000
C	-0.72461900	-1.25507700	-0.06341300
C	1.44923800	0.00000000	-0.06341300
C	-0.72461900	1.25507700	-0.06341300
H	-1.75619900	1.27597400	0.28919900
H	-0.74364700	1.28803400	-1.15521700
H	-0.22692600	2.15890000	0.28919900
H	1.48729400	0.00000000	-1.15521700
H	1.98312600	-0.88292600	0.28919900
H	0.00000000	0.00000000	1.50700300

***n*-C_5H_{12}**
E = -2761.2
H = -2654.9
N_{imag} = 0

C	0.31552700	-0.20904100	2.53665200
C	0.31552700	-0.20904100	-2.53665200
H	-0.43872400	1.36247600	-1.27104300
H	-1.42279600	-0.08328300	-1.27070900
C	0.30364700	-0.20035600	0.00000000
C	-0.38885100	0.27073400	-1.27319200
C	-0.38885100	0.27073400	1.27319200
H	0.35508800	-1.29373300	0.00000000
H	-0.19225400	0.13634600	-3.43640900
H	0.35202400	-1.29872900	-2.56999300
H	1.34267600	0.15658900	-2.57038000
H	-1.42279600	-0.08328300	1.27070900
H	-0.43872400	1.36247600	1.27104300
H	-0.19225400	0.13634600	3.43640900
H	1.34267600	0.15658900	2.57038000
H	0.35202400	-1.29872900	2.56999300
H	1.33953800	0.15324700	0.00000000

***i*-C_5H_{12}**
E = -2762.1
H = -2656.1
N_{imag} = 0

C	1.78601800	0.86216900	0.53826300
H	-2.25839700	-1.53578800	0.08494100
H	-0.74137100	-2.43063300	0.21169700
H	-1.13902400	-1.57167400	-1.27698400
C	-0.50630000	-0.27199700	0.33165900
C	-1.20268700	-1.52668100	-0.18732700
C	0.98482100	-0.31409600	-0.00905000
C	-1.18309100	0.97848900	-0.22406600
H	-2.25369400	0.96501700	-0.01800300

H	-1.05226900	1.02871000	-1.30799200
H	-0.77354200	1.89111200	0.20692500
H	1.09224300	-0.35834200	-1.09749600
H	1.40286700	-1.24630900	0.37824700
H	2.85231000	0.72688000	0.36120100
H	1.63684900	0.96504600	1.61455800
H	1.49231500	1.80098600	0.07016700
H	-0.59832000	-0.25432100	1.42258200

***i*-C_6H_{14}**
E = -3269.2
H = -3144.4
N_{imag} = 0

C	1.75758600	0.88026000	0.65402800
H	-2.24669200	-1.55505600	0.00060500
H	-0.72683700	-2.43351800	0.19250600
H	-1.06367200	-1.56745800	-1.30698600
C	-0.52343200	-0.27313400	0.33953400
C	-1.17944500	-1.53184700	-0.22131000
C	0.98225000	-0.29675500	0.07166700
C	-1.18777100	0.97344600	-0.24011100
H	-2.26779900	0.94167900	-0.09360800
H	-0.99824600	1.03759000	-1.31458300
H	-0.81655700	1.88718100	0.22201400
H	1.14696500	-0.33669000	-1.01125000
H	1.39566600	-1.22537400	0.47715400
C	3.26373900	0.71942500	0.48110300
H	1.51658100	0.97910800	1.71603800
H	1.43742300	1.80779300	0.17649900
H	3.80852500	1.57059900	0.88784700
H	3.52407300	0.62886200	-0.57442000
H	3.61921800	-0.17936500	0.98660200
H	-0.66957900	-0.26500500	1.42451300

***n*-C_7H_{16}**
E = -3775.5
H = -3631.6
N_{imag} = 0

C	1.27219400	-0.31085300	0.08649900
C	-2.54504900	0.49770200	-0.15391300
C	-3.80787200	-0.34916800	-0.05007300
C	2.54479200	0.52174500	-0.01612000
C	-0.00015400	0.50995500	-0.08438900
C	-1.27206600	-0.32289400	0.01754600
C	3.80808200	-0.31315800	0.15636800
H	1.29716500	-1.10201800	-0.66930100
H	1.24954800	-0.81665400	1.05677900
H	-2.56724200	1.28678800	0.60186900
H	-2.51831900	1.00221400	-1.12298600
H	-4.70741000	0.25244000	-0.17506200
H	-3.86607100	-0.84078800	0.92194000
H	-3.81652000	-1.12742800	-0.81435400
H	2.56586400	1.02602900	-0.98545400
H	2.51863000	1.31098000	0.73938000
H	-0.02435900	1.30069900	0.67172900
H	0.02375500	1.01576600	-1.05452300
H	-1.29722000	-0.82879300	0.98771400
H	-1.24868700	-1.11400000	-0.73836700
H	3.86534700	-1.09138100	-0.60585100

H	3.81825100	-0.80410800	1.13040400
H	4.70734300	0.29692600	0.07983200

C_7H_{16}
E = -3775.3
H = -3631.5
N_{imag} = 0

C	2.41149000	0.95096700	0.52807500
C	-2.03479100	-1.62016200	0.49853300
H	-0.02361500	-2.37435400	0.41826800
H	-0.51973200	-1.53810400	-1.02937900
C	0.13684700	-0.24616800	0.56796800
C	-0.57858400	-1.50338800	0.06182500
C	1.58834400	-0.25036400	0.07626700
C	-0.60880000	1.04720000	0.21395000
H	-1.60005800	1.01979000	0.66950100
C	-0.74744200	1.30349500	-1.28387000
H	-0.09511400	1.89016000	0.67916500
H	1.60002800	-0.31245100	-1.01524600
H	2.06281700	-1.16784100	0.43226600
H	3.46251700	0.82432900	0.27122500
H	2.34504900	1.08435800	1.60938000
H	2.06785700	1.87289600	0.05880800
H	-2.12792300	-1.50259000	1.57970300
H	-2.44676300	-2.59265000	0.23169300
H	-2.65773700	-0.85955500	0.02772500
H	0.22682900	1.38334200	-1.76765500
H	-1.28401500	2.23305200	-1.46997700
H	-1.29759400	0.50143600	-1.77747500
H	0.16288100	-0.30374700	1.66271100

$C_{10}H_{22}$
E = -5296.6
H = -5096.4
N_{imag} = 0

C	2.48038500	0.91423000	0.47384300
C	-1.95088200	-1.70608300	0.73904900
H	0.04960500	-2.43145900	0.47602300
H	-0.56661700	-1.54602600	-0.89594800
C	0.21655300	-0.30603700	0.69036100
C	-0.53391500	-1.54830800	0.19831500
C	1.61710400	-0.27572900	0.06898400
C	-0.56618900	0.99379100	0.46438800
H	-1.51262700	0.93987700	1.00828300
C	-0.85478700	1.32833500	-0.99581800
H	-0.01724400	1.82424600	0.91545800
H	1.53361700	-0.30254200	-1.02224300
H	2.13459900	-1.19817500	0.34982300
C	3.91509800	0.77848600	-0.02350000
H	2.47382900	1.01253900	1.56296600
H	2.04855300	1.83640000	0.07898000
H	-1.93763900	-1.59650700	1.82703400
C	-2.56264000	-3.05090900	0.36311500
H	-2.58469700	-0.90309200	0.35656100
H	0.08347700	1.40330700	-1.55079200
C	-1.63310700	2.63169400	-1.13756800
H	-1.42079200	0.51528900	-1.45684600
H	4.52033800	1.64112200	0.25278800
H	3.94004700	0.68635200	-1.11033200

H	4.38832500	-0.11139400	0.39345200
H	-1.97469500	-3.87332300	0.77254000
H	-2.59020500	-3.17321100	-0.72067200
H	-3.58141900	-3.14828400	0.73647200
H	-1.07438100	3.46488100	-0.70876300
H	-2.58802500	2.57133100	-0.61341300
H	-1.83813200	2.86761300	-2.18116800
H	0.34353100	-0.41036600	1.77428400
